# Seasonal Dynamics of Bat Activity in the Bosphorus Region (Istanbul, Türkiye): Acoustic Evidence for a Potential Migration Corridor Between Europe and Asia

**DOI:** 10.3390/ani16213346

**Published:** 2026-10-23

**Authors:** Tuğçe Nur İlbaş Gür, Emrah Çoraman

**Affiliations:** Eurasia Institute of Earth Sciences, Istanbul Technical University, 34467 Istanbul, Türkiye; ilbas15@itu.edu.tr

**Keywords:** bat migration, passive acoustic monitoring, *Pipistrellus nathusii*, *Nyctalus*, Bosphorus, migration corridor, wind direction, phenology, wind energy

## Abstract

Long-distance migratory bats may face concentrated risks where movements funnel through geographic corridors, yet migration in southeastern Europe remains poorly documented. Istanbul and the Bosphorus are internationally recognized as a major migration bottleneck for birds, but their importance for bats has not been systematically investigated. We used passive acoustic monitoring at four stations along the Bosphorus from April to November 2022 to characterize bat activity and species occurrence, with detailed seasonal and meteorological analyses focused on *Pipistrellus pipistrellus*, *P. nathusii*, and the *Nyctalus noctula/N. leisleri* acoustic group. Across 3613 station-hour observations and 259,654 bat passes, at least twelve species were identified, including the globally Vulnerable *N. lasiopterus*. *Pipistrellus pipistrellus* was active throughout most of the season, whereas 84 to 93% of *P. nathusii* passes occurred after mid-August, peaking synchronously at all four stations between 26 and 29 September. Season-specific wind associations were weak, not supported by cross-validation, and shared by the resident *P. pipistrellus*, indicating weather effects on local activity rather than directed movement. These patterns are consistent with seasonal bat migration through the Istanbul–Bosphorus region and support the hypothesis that the strait forms a migration corridor, highlighting its potential importance for conservation and wind-energy planning.

## Simple Summary

Some European bats travel more than two thousand kilometres each year between their summer and winter areas. Where these journeys funnel through narrow landscapes, bats become easier to disturb; wind turbines placed along a busy route, for example, can kill large numbers of migrating bats in a single season. The Bosphorus Strait in Istanbul is famous as a bottleneck for migrating birds, but no one had asked whether bats also pass through it. We placed ultrasonic recorders at four sites in Istanbul, from a dense city park to a northern forest, and recorded bat calls on regular nights from April to November 2022. We detected at least twelve species, including the rare greater noctule. Migratory species were scarce in summer but appeared in large numbers in late September at all four sites within the same few nights, a pattern consistent with seasonal passage rather than year-round local activity. Our findings indicate that migratory bats move through Istanbul in autumn, support the hypothesis that the Bosphorus forms a migration route for bats, and identify seasonal windows during which additional precautions may be warranted for human activities along the strait, including wind-energy development.

## 1. Introduction

Seasonal migration is one of the most widespread movement strategies in animals, and migratory species link populations, communities, and ecosystems across continents [1,2]. Because migrants depend on a sequence of sites and routes, they are particularly vulnerable where movements are funnelled through geographic corridors and bottlenecks, which can concentrate a large proportion of a population within a small area during brief periods of the year [3]. The rapid global expansion of wind energy has added a new pressure on such concentrated movements, and collision and displacement effects on birds and bats are among the best-documented wildlife impacts of renewable-energy infrastructure [4,5]. Among mammals, bats are particularly affected.

Long-distance migration can expose bats to multiple anthropogenic pressures, particularly where movements are concentrated along geographic corridors. Habitat loss and fragmentation can disrupt commuting and stopover areas, while artificial light may alter movement through otherwise suitable corridors [6]. Wind-energy development poses an additional and particularly important threat because long-distance migratory species such as Nathusius’ pipistrelle (*Pipistrellus nathusii*), the common noctule (*Nyctalus noctula*), and Leisler’s bat (*N. leisleri*) are disproportionately represented among turbine fatalities [7]. High mortality involving these species has been documented at wind farms in both Eastern and Central Europe [8,9]. Where migration is spatially concentrated, development along movement corridors may therefore expose bats originating from geographically widespread populations, making the identification of migration routes and periods an important conservation priority.

Knowledge of European bat migration, however, remains geographically uneven. Decades of banding, acoustic surveys, offshore monitoring, and more recently biologging have provided considerable information from northern and western Europe [10,11,12,13,14,15]. These studies show that several long-distance migrants move broadly along a northeast-to-southwest axis, with individual *P. nathusii* travelling more than 2000 km [16,17]. Recent tracking studies have further demonstrated that migratory bats can undertake long nocturnal flights and adjust their movements to atmospheric conditions [18]. In contrast, migration routes through southeastern Europe, the Black Sea region, and western Asia remain much less documented, and systematic acoustic studies of bat migration at the Turkish Straits are lacking. The straits lie along the broad northeast-to-southwest migration axis and may therefore funnel movements between Europe and Asia. Consequently, potentially important connections between European breeding populations and areas farther south and east are still poorly understood.

Understanding when and under which conditions migration occurs is also important for conservation. Bat activity and migratory movement are strongly influenced by temperature and wind, and passage can become concentrated within relatively narrow seasonal and meteorological windows [13,19,20]. Such predictability creates opportunities for targeted mitigation at wind-energy facilities. Curtailment can be restricted to periods when collision risk is highest, potentially reducing mortality while limiting unnecessary losses in energy production [21]. This requires information not only on migration phenology, but also on the environmental conditions associated with migratory activity. Collision risk is not restricted to bats in active passage, because bats are also killed while foraging or engaging in social and mating behaviour around turbines [22,23,24,25]. Wind direction may be especially informative because migratory bats can adjust their movements in relation to prevailing winds, and the direction of favourable wind support should change between spring and autumn. For species such as *P. nathusii*, which generally migrate northeastward in spring and southwestward in autumn, a corresponding seasonal shift in wind associations would therefore provide stronger evidence of directed movement than seasonal timing alone. Although wind-assisted migration is increasingly documented in bats, the same tracking work also shows that individuals do not move under supportive winds alone and may travel against the prevailing direction [13,18,26], and seasonal and regional differences in these wind associations remain poorly resolved.

Türkiye lies at the centre of this geographic gap. Situated between southeastern Europe, the Black Sea, and western Asia, it may lie along the seasonal migration routes of European bats, yet bat research in the country has largely focused on taxonomy, distribution, roosts, and phylogeography [27,28,29], while seasonal activity and large-scale movements remain poorly studied. Acoustic monitoring has so far been applied in relatively few ecological studies, and these have generally not focused on systematic assessment of bat migration [30,31,32]. *Pipistrellus nathusii* has been recorded over the western Black Sea [33], and a recent acoustic survey on the northern Marmara coast documented a pronounced autumn peak in *P. nathusii* activity [34], suggesting that long-distance movements extend through this area. Within this broader landscape, the Bosphorus forms a narrow geographic connection between Europe and Anatolia and is already recognized as one of the world’s major migration bottlenecks for soaring birds [35]. Whether it also contributes to concentrating bat movements has not been investigated. This knowledge gap is increasingly pressing because wind-energy development in the region is accelerating. By January 2026, installed wind capacity in Türkiye had reached approximately 15,900 MW, of which approximately 7000 MW was located in the Marmara Region, the largest share of any region, including approximately 950 MW in Istanbul Province [36]. Installed capacity in Istanbul has more than doubled since 2021, the Thracian provinces of Tekirdağ, Kırklareli, and Edirne hold the largest pipelines of pre-licensed wind projects [37,38], and offshore wind zones have been proposed in the Sea of Marmara [34].

Here, we used passive acoustic monitoring at four sites in Istanbul between April and November 2022 to investigate whether seasonal bat activity is consistent with migration through the Istanbul–Bosphorus region. We focused on *P. pipistrellus* as a widespread comparison species, *P. nathusii* as the principal long-distance migrant, and the *N. noctula/N. leisleri* acoustic group as a second long-distance migratory group; the latter two species overlap in their echolocation calls and were therefore analysed together. We examined spatial and seasonal variation in activity and its association with temperature, wind, and other environmental conditions, and we tested whether wind responses differed between spring and autumn. If seasonal activity reflects migratory movement through the region rather than foraging by bats of the local, resident population, we predicted that peak activity should track supportive winds along opposite axes in the two seasons, reversing between the northeastward spring and southwestward autumn movements. By identifying both the timing and the environmental conditions associated with seasonal movements, we aim to improve understanding of a poorly studied part of the Eurasian bat migration system and to support the spatial and temporal targeting of conservation and mitigation efforts for migratory bats.

## 2. Materials and Methods

### 2.1. Study Area and Acoustic Data Collection

**Figure 1:**
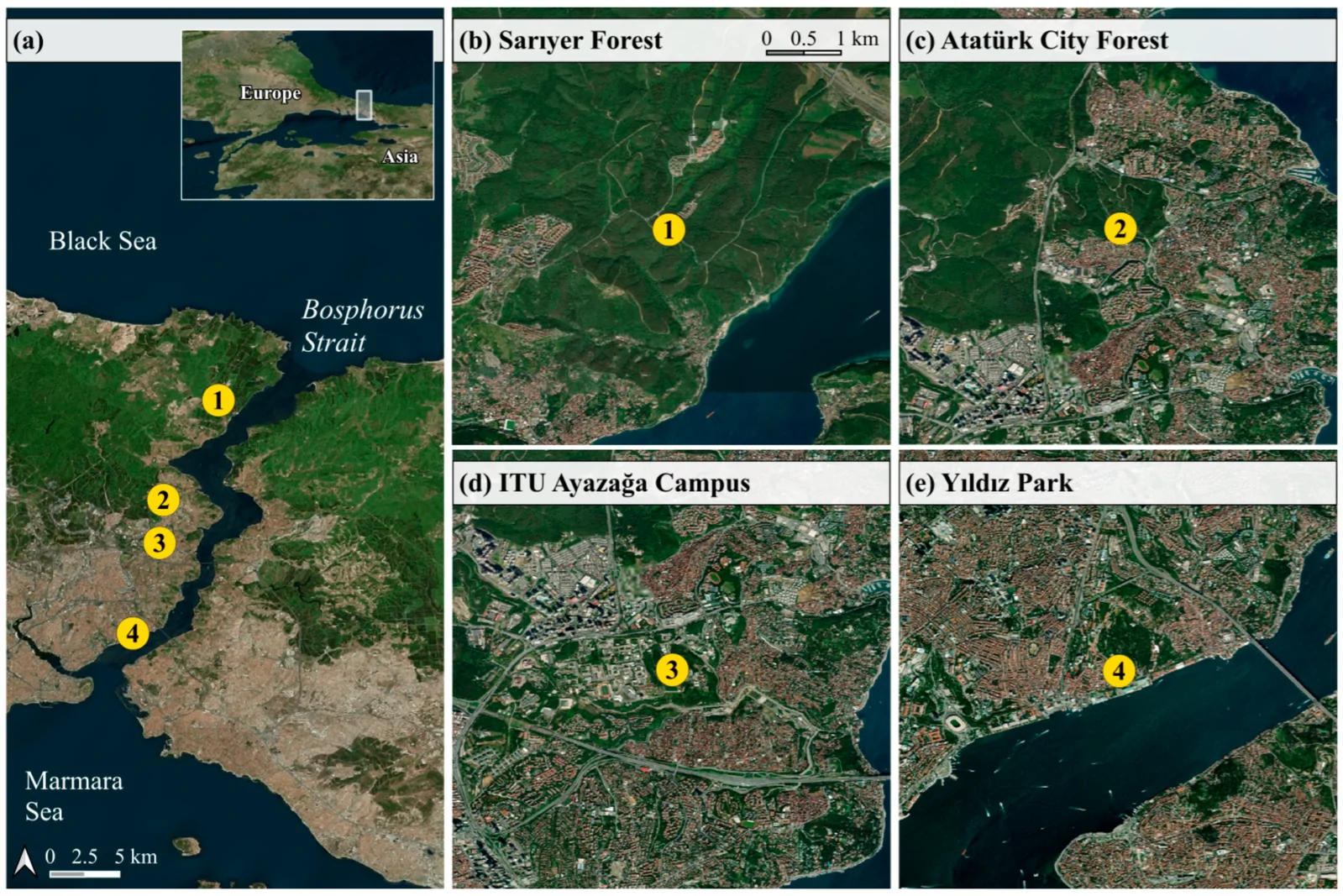
Study area and locations of the four passive acoustic monitoring stations along the Bosphorus in Istanbul, Türkiye. (**a**) Regional overview showing the position of the study area between the Black Sea and the Sea of Marmara, with the inset indicating its location between Europe and Asia. Monitoring stations are numbered from north to south: (1) Sarıyer Forest, (2) Atatürk City Forest, (3) Istanbul Technical University Ayazağa Campus, and (4) Yıldız Park. Panels (**b**–**e**) show the local setting of each monitoring station. Satellite imagery: Esri World Imagery.

Acoustic recordings were collected at four locations along the Bosphorus, representing a gradient from forested to highly urbanized settings (Figure 1; Table S1). From north to south, these were: (1) Sarıyer Forest (41.196° N, 29.070° E), a forested peri-urban site in the northern forest belt; (2) Atatürk City Forest (ACF; 41.132° N, 29.034° E), an urban forest with mixed woodland and recreational clearings; (3) Istanbul Technical University (ITU) Ayazağa Campus (41.104° N, 29.032° E), a university campus with mixed built, wooded, and open green areas; and (4) Yıldız Park (41.046° N, 29.014° E), a historic urban park with mature trees and landscaped green space in a densely urbanized area.

At each location, a Wildlife Acoustics Song Meter Mini Bat (first generation; Wildlife Acoustics, Maynard, MA, USA) detector was deployed at least 2 m above ground, with the ultrasonic microphone oriented upward. Straight-line distances from the stations to the shoreline of the Bosphorus were 1.3 km (Sarıyer Forest), 2.0 km (ACF), 1.9 km (ITU), and 0.4 km (Yıldız Park). Detectors operated from 30 min before sunset to 30 min after sunrise and recorded in full spectrum at a sample rate of 256 kHz, with a minimum trigger frequency of 6 kHz, a trigger window of 3 s, and a maximum recording duration of 15 s. A bat pass was defined as a recording file containing at least two echolocation calls of the same taxon. Recordings were collected from April to November 2022.

### 2.2. Meteorological and Lunar Phase Data

Meteorological data were obtained from the NASA Langley Research Center POWER Project [39]. Four parameters were retrieved for each location: wind speed and direction at 10 m above ground level, air temperature at 2 m above ground level, and precipitation. NASA POWER provides gridded estimates at a spatial resolution of 0.5° latitude × 0.625° longitude. ACF, ITU, and Yıldız Park fall within the same grid cell and therefore share identical hourly weather series, whereas Sarıyer Forest lies in the adjacent cell; between-station differences in weather were therefore represented only between Sarıyer Forest and the remaining stations. Moon phase for each recording date was determined using the R package lunar [40]. Hourly meteorological observations were matched to acoustic observations by station and corresponding hourly timestamp. Hourly data were retrieved in UTC for both grid cells and converted to local civil time (UTC + 3), the time standard of the detector timestamps, before matching.

### 2.3. Acoustic Processing and Taxonomic Assignment

Not every recorded night was processed. Nights were selected for processing to achieve an approximately uniform distribution of sampling effort across the monitoring season at each station. Nights selected for acoustic analysis were spaced at station-specific intervals, with median intervals of 1–2 days and mean intervals of 2.0–3.4 days across stations. Sampling was approximately nightly during spring and autumn and less intensive in summer, when the median interval between processed nights was 4 days at ACF, ITU, and Yıldız Park and 1 day at Sarıyer Forest. The longest gaps occurred at ITU (27 April–10 June) and ACF (13 May–10 June). These gaps arose because the detector at ACF failed shortly after deployment, and the ITU unit was alternated between the two stations from 22 April to 9 June 2022. After a replacement detector was obtained, recording continued at both stations without further interruption. Nights with precipitation totals exceeding 18 mm (NASA POWER corrected precipitation, summed over the night) were excluded from processing, because preliminary inspection of the recordings showed no significant bat activity above approximately this level. Consequently, heavy-rainfall conditions were poorly represented in the analysed dataset, and any estimated effect of precipitation should be interpreted only within the restricted range of conditions sampled.

Acoustic processing was conducted in two stages. Recordings were first processed using Kaleidoscope Pro 5.4.9 [41], which provided provisional species classifications using its automated classifier. All recordings were subsequently examined manually through visual inspection of spectrograms in BatExplorer v2.2.6.0 (Elekon AG, Lucerne, Switzerland) and Kaleidoscope Pro v5.6.8 [41]. Provisional identifications were verified and corrected where necessary, low-amplitude calls missed by the automated classifier were identified where possible, and recordings containing more than one species or individual were recorded accordingly.

Manual identification followed Barataud [42], Dietz and Kiefer [43], and Russ’s bat-call identification guides [44,45]. Recordings were assigned to the lowest taxonomic level that could be identified confidently from their acoustic characteristics. Where echolocation calls showed substantial interspecific overlap, records were retained as acoustic groups rather than forcing species-level identification. Calls attributable to *Myotis*, for example, were combined as *Myotis* spp. Similarly, because the echolocation calls of *Nyctalus noctula* and *N. leisleri* overlap considerably, these species were pooled as the *N. noctula/N. leisleri* acoustic group for activity analyses. Where either species could be identified confidently, the species-level record was retained for occurrence summaries, but all corresponding records were combined for modelling of the focal acoustic group.

Because the echolocation calls of *Nyctalus lasiopterus* overlap with those of *Tadarida teniotis* and *N. noctula*, sequences were assigned to *N. lasiopterus* only when they exhibited a combination of characteristic acoustic features, including a frequency of maximum energy (FME) of approximately 14–17 kHz and an initial frequency-modulated component followed by a long quasi-constant-frequency tail. Calls with higher FME (typically above 17–18 kHz) were attributed to *N. noctula*, whereas long, narrowband calls with lower FME (approximately 10–13 kHz) were attributed to *T. teniotis* [42,45]. Representative spectrograms are provided in Figure S6.

Acoustic activity was quantified as the number of bat passes recorded for each taxon. Following identification, pass counts were summed within each station, night, and hourly interval to produce the hourly response variables used in subsequent analyses. Each night was dated by the evening on which it began (i.e., the date of sunset), so that recordings made after midnight were assigned to the preceding calendar date; this night date defined both the station-night identifier and the calendar-night random effect used in the models (Section 2.4 and Section 2.5). The complete processed dataset comprised 3613 station-hour observations derived from the underlying recording files. Bat-pass counts were treated as an index of acoustic activity and not as a measure of the number of individual bats, because the same individual may be detected repeatedly within or among recording intervals.

Particular consideration was required for *Pipistrellus nathusii* because its echolocation calls overlap strongly with those of *P. kuhlii*, and the two species cannot always be separated reliably using echolocation calls alone [44]. Social calls can provide more reliable discrimination, but were not consistently available in the recordings. Regional evidence nevertheless suggests that *P. nathusii* is likely to constitute the majority of calls assigned to this acoustic category. In a Ph.D. thesis examining bat fatalities at wind farms in Türkiye, Şaşmaz [46] reported *P. nathusii* as the second most frequently identified carcass species at wind farms in Tekirdağ Province, near Istanbul, whereas *P. kuhlii* was not recorded; these findings were also discussed by Çoraman [34] in relation to seasonal acoustic activity on the northern Marmara coast. In addition, *P. nathusii* has been identified on several occasions in Istanbul by the second author (E. Çoraman, unpublished observations), while confirmed regional records of *P. kuhlii* remain comparatively scarce. The regional occurrence of *P. nathusii* has also been confirmed by recent records [47,48]. We therefore treated this overlapping acoustic category operationally as *P. nathusii* in the focal analyses, while acknowledging that some *P. kuhlii* calls may have been included.

For species-richness summaries, recordings were assigned to the lowest taxonomic level that could be identified confidently. Unresolved acoustic groups containing species already represented by species-level records were not counted as additional species. *Myotis* spp. was conservatively counted as representing a minimum of one additional species, and *Plecotus* sp. was treated in the same way, although the group may contain several species. Reported richness values should therefore be regarded as minimum estimates.

### 2.4. Full-Season Activity Models

Hourly bat-pass counts were modelled separately for the three focal taxa, *Pipistrellus pipistrellus*, *P. nathusii*, and the *N. noctula/N. leisleri* acoustic group, using generalized additive models (GAMs) with a negative-binomial error distribution and log link. The negative-binomial distribution was used to accommodate overdispersion in the acoustic count data, which contained many zero or low-count hours together with comparatively few hours of very high activity. Models were fitted using bam() in the mgcv package [49] in R v4.3.3 [50]. The complete R code used for data processing, model fitting, and figure generation is provided as an R Markdown document (Supplementary File S1).

Full-season models were designed to separate seasonal phenology from within-night and environmental variation in activity. Site was included as a fixed factor to account for overall differences in activity among the four monitoring stations. Seasonal phenology was represented by separate smooth functions of day of year for each site, allowing the shape and timing of seasonal activity to vary among stations. Additional predictors were hours after sunset, air temperature, 10 m wind speed, 10 m wind direction, lunar phase, and precipitation. A random intercept for calendar night was also included. Hours after sunset was used rather than clock time to account for seasonal changes in sunset time.

Continuous predictors were fitted using penalized smooth functions, with the complexity of each smooth summarized by its effective degrees of freedom (EDFs). Wind direction was treated as a circular variable and fitted using a cyclic cubic regression spline so that directions near 0° and 360° were treated as adjacent. Lunar phase was likewise represented as a continuous cyclic variable and fitted with a cyclic cubic spline. Basis dimensions were checked using standard mgcv diagnostics, and concurvity among smooth terms was examined to identify potentially redundant predictor relationships.

To evaluate whether seasonal phenology differed among monitoring stations, the full model containing site-specific smooths of day of year was compared with an otherwise equivalent model containing a single seasonal smooth shared among sites. Model support was compared using Akaike’s Information Criterion (AIC), with lower AIC indicating better support, and by five-fold grouped cross-validation.

Complementary presence–absence models were fitted separately for each focal taxon using a binomial error distribution and logit link. These models used the same predictor structure as the count models and were treated as sensitivity analyses to determine whether the main patterns were also evident in the probability of acoustic detection rather than being driven solely by variation in the number of recorded passes.

### 2.5. Temporal Autocorrelation and Model Evaluation

Because successive hourly observations collected within the same station-night were temporally ordered and potentially autocorrelated, each combination of monitoring station and date was assigned a unique station-night identifier. Observations were ordered chronologically within each station-night, and lag-1 residual autocorrelation was estimated from initial negative-binomial models using Pearson residuals. Final models were then refitted using the rho and AR.start arguments in mgcv::bam() with discrete = TRUE, applying an AR(1) working-residual correction. The beginning of each station-night was specified as the start of a new autocorrelation series. Station-night identity was used only to define these independent temporal series and was not included as a random-effect term.

Model performance was evaluated using AIC, deviance explained, and residual diagnostics. For non-Gaussian families, the AR(1) correction in mgcv::bam() is a working-correlation approximation that is not reflected in AIC. AIC-based model comparisons were therefore supported throughout by five-fold grouped cross-validation, with all hourly observations from the same calendar night assigned to the same fold, summarized as root mean squared error (RMSE) and mean absolute error (MAE). Because the stations share weather conditions, observations from the same night are not independent across stations; calendar night was therefore included as a random intercept (s(night, bs = “re”) in mgcv) in all count models, which accounts for conditions shared by all stations on the same night.

For the complementary presence–absence models, predictive discrimination was assessed using the area under the receiver operating characteristic curve (AUC). Model diagnostics for the primary count models included examination of residual autocorrelation, dispersion, basis adequacy, and concurvity among smooth terms.

### 2.6. Seasonal Wind-Direction Analyses

To test whether associations between bat activity and wind direction differed between the spring and autumn windows, wind effects were analysed separately for spring and autumn. Because independently established seasonal windows were not available for the study region, the seasonal intervals were defined a priori for exploratory analysis rather than estimated from the acoustic data. Spring was defined as observations before 1 June, whereas autumn was defined as observations from 14 August to 9 November. The autumn window ended on 9 November because this was the last night on which more than one station was sampled; the final sampled night (10 November) was recorded only at Sarıyer Forest and was therefore not included in the seasonal-window analyses. These boundaries are broadly consistent with the spring and autumn migration periods reported for *P. nathusii* and *Nyctalus* species in Europe [11,13].

Wind availability within each seasonal window was first summarized independently of bat activity. Wind direction was divided into 24 sectors of 15°, and the number of hourly observations within each sector was calculated to assess directional coverage and identify poorly represented wind conditions.

For *P. nathusii*, the *N. noctula*/*N. leisleri* acoustic group, and *P. pipistrellus*, which was included as a resident comparison, negative-binomial models with the calendar-night random effect were fitted to the spring–autumn window subset of the data. To test whether the relationship between activity and wind direction differed between spring and autumn, two alternative model structures were compared. The first included a single circular wind-direction smooth shared between seasons, whereas the second allowed separate circular smooths for spring and autumn. Apart from the wind-direction structure, the models contained the same covariates, including site, within-season temporal variation, hours after sunset, temperature, wind speed, lunar phase, and precipitation. Wind direction was fitted using cyclic cubic regression splines to preserve continuity between 0° and 360°.

The common and season-specific formulations were compared using AIC and deviance explained, together with five-fold grouped cross-validation. Because the AR(1) correction is not reflected in AIC, a season-specific formulation was regarded as supported only if it also improved cross-validated prediction. In addition, a seasonal difference in the migratory taxa was interpreted as related to migration only if it was not also present in the resident *P. pipistrellus*. All seasonal wind analyses were regarded as exploratory.

### 2.7. Migration-Relative Wind Support

Wind conditions were additionally expressed relative to the expected direction of seasonal migration. Spring movement was assumed to be toward approximately 45° (northeast), whereas autumn movement was assumed to be toward approximately 225° (southwest). These bearings represent the broad northeast-to-southwest migration axis described for long-distance migratory bats in continental Europe [10,51]. This axis also corresponds to the main direction of seasonal passage of migratory birds through the Bosphorus [35].

NASA POWER wind direction follows the meteorological convention and therefore indicates the direction from which the wind originates. Wind direction was first converted to the corresponding direction of airflow, after which wind speed was decomposed into components parallel and perpendicular to the assumed migration axis. The parallel component was expressed as tailwind support. Positive values indicate winds supporting movement in the assumed migration direction, negative values indicate opposing headwinds, and values close to zero indicate little or no wind support along the migration axis. The perpendicular component was retained as crosswind, with positive and negative values representing winds acting from opposite sides of the migration axis. Thus, winds originating from the southwest provide tailwind support for northeastward spring movement, whereas winds originating from the northeast provide support for southwestward autumn movement.

The same seasonal migration axes were applied to *P. nathusii* and the *N. noctula/N. leisleri* acoustic group, and to *P. pipistrellus* as a resident comparison. For *P. nathusii*, this axis is consistent with its well-documented seasonal movements across Europe. *N. noctula* and *N. leisleri* also migrate broadly along a northeast-to-southwest axis [10,51], but because a single migration bearing specific to the pooled acoustic group has not been established, the migration-relative analysis for this group was regarded as exploratory.

The circular wind-direction models described in Section 2.6 and the migration-relative models addressed complementary questions. The circular models estimated the wind directions associated with bat activity without assuming a movement direction. In contrast, the migration-relative models tested whether activity varied with the degree of wind support along the expected seasonal migration axis. Because the tailwind and crosswind components jointly incorporate both wind speed and direction, raw wind speed and wind direction were not included separately in these models.

Migration-relative models were fitted to the spring and autumn periods defined in Section 2.6. Models included site, within-season temporal variation, hours after sunset, temperature, tailwind, crosswind, lunar phase, and precipitation. To test whether the relationship between tailwind support and activity differed between the two seasonal windows, a model containing a common tailwind smooth for spring and autumn was compared with an otherwise equivalent model allowing separate seasonal tailwind smooths. Model support was evaluated using AIC and deviance explained, together with grouped cross-validation.

Predicted tailwind responses were generated across the observed range of wind-support values. Because predictions near the extremes of the distribution may be based on relatively few observations, the distributions of hourly observations and positive detection hours were examined alongside the fitted response curves. Portions of the curves with limited observational support were interpreted cautiously, and direct comparisons between spring and autumn responses were restricted to wind-support values represented in both seasons. Specifically, curves were shown and interpreted only within the range supported by at least 30 hourly records and at least five hours with activity per 1 m s^−1^ bin of tailwind support (or per 15° sector of wind direction). For tailwind support, this range was −6 to +4 m s^−1^ in spring and −5 to +10 m s^−1^ in autumn (−5 to +9 m s^−1^ for the *N. noctula*/*N. leisleri* acoustic group).

## 3. Results

### 3.1. Sampling Effort and Species Richness

Acoustic recordings were collected at the four monitoring stations on nights between 7 April and 10 November 2022 (the last recordings ending on the morning of 11 November). The dataset comprised 3613 hourly detector records from 315 station-nights spanning 165 calendar nights. Survey effort varied among stations, with the greatest coverage at Sarıyer Forest (105 nights), followed by Yıldız Park (74 nights), ACF (72 nights), and ITU (64 nights). Across all stations, 259,654 bat passes were recorded. *Pipistrellus pipistrellus* was by far the most frequently recorded taxon (212,012 passes; 81.7% of total activity), followed by *P. nathusii* (40,647 passes; 15.7%) and the *N. noctula/N. leisleri* acoustic group (6368 passes; 2.5%).

**Figure 2:**
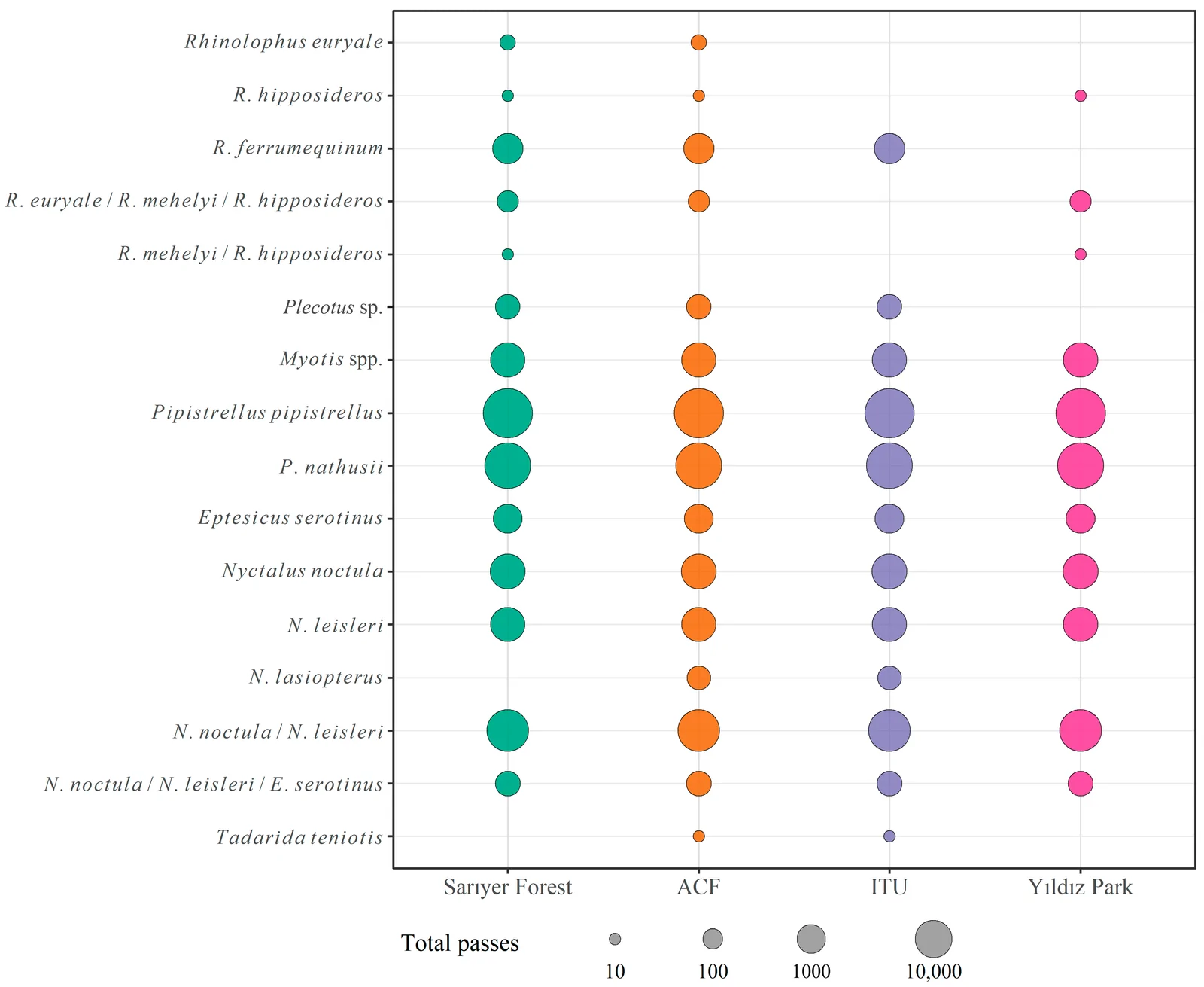
Occurrence of acoustic taxa across monitoring stations. Rows show species or acoustic groups, and columns show monitoring stations. A bubble indicates that the taxon was detected at that station; bubble area is scaled to the log10-transformed number of bat passes for that taxon at that station.

At least twelve species were recorded across the four monitoring stations. Ten were identified to species level: *Pipistrellus pipistrellus*, *P. nathusii*, *Nyctalus noctula*, *N. leisleri*, *N. lasiopterus*, *Eptesicus serotinus*, *Rhinolophus ferrumequinum*, *R. euryale*, *R. hipposideros*, and *Tadarida teniotis* (Figure 2; Table S2). Additional detections were assigned to *Plecotus* sp., *Myotis* spp. and acoustically unresolved species groups, indicating that actual richness was likely higher. Five species (*P. pipistrellus*, *P. nathusii*, *N. noctula*, *N. leisleri*, and *E. serotinus*) were detected at all four stations, whereas several other species showed more restricted distributions. *Rhinolophus ferrumequinum* was recorded predominantly at Sarıyer Forest, while *Plecotus* sp. occurred mainly at ACF. *Nyctalus lasiopterus* was recorded on 11 nights (22 passes; 10 nights at ACF, one at ITU; Figure S6), while *R. euryale* was detected at ACF and Sarıyer Forest. Seasonal detections of less frequently recorded taxa are shown in Figure S5.

Observed richness was at least 12 species at ACF, followed by at least 10 species each at ITU and Sarıyer Forest, and at least seven species at Yıldız Park, when *Myotis* spp. and *Plecotus* sp. are each counted as representing a minimum of one additional species.

### 3.2. Spatial and Seasonal Activity Patterns

**Figure 3:**
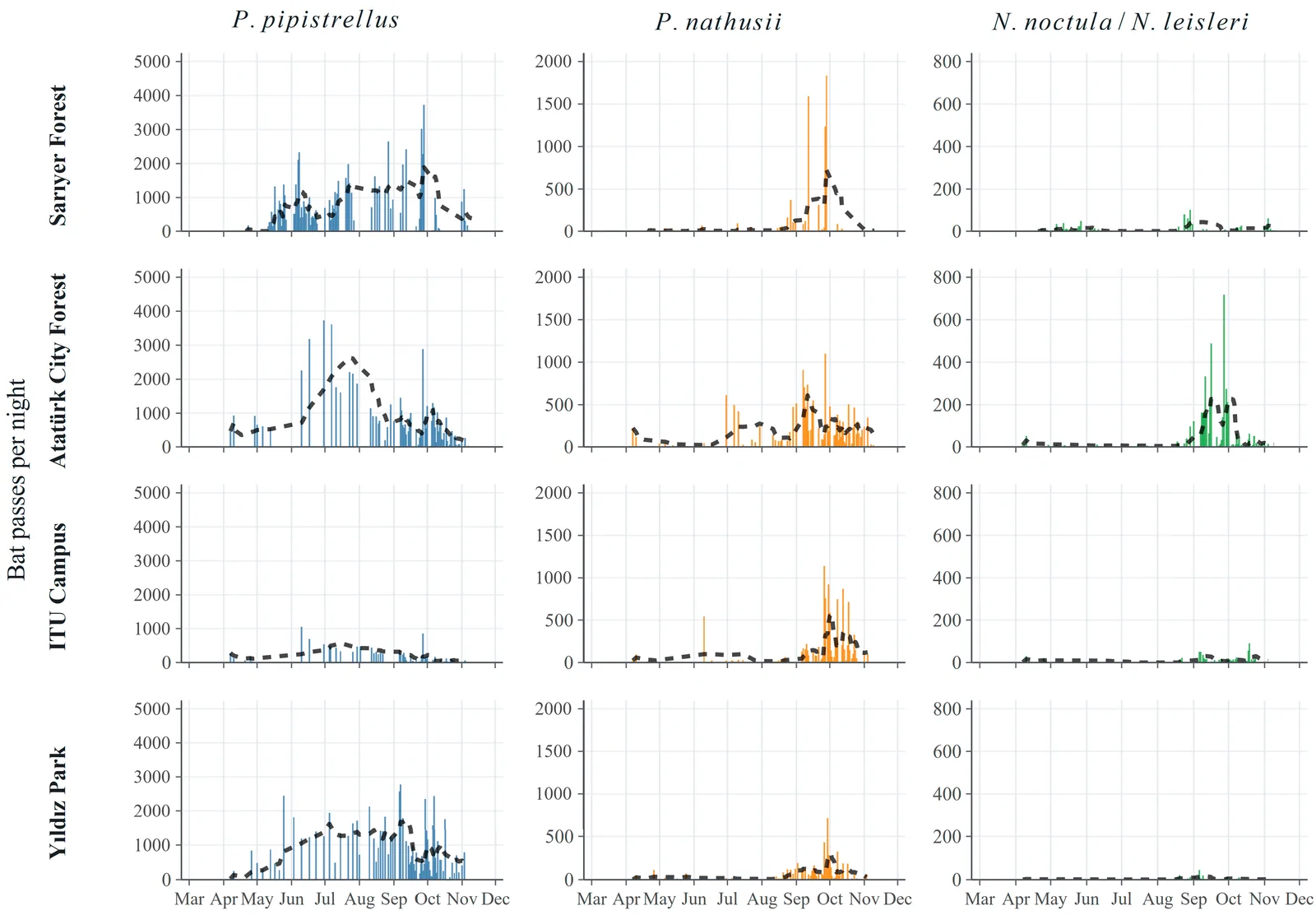
Seasonal nightly activity of focal bat taxa across monitoring stations. Bars show nightly summed acoustic activity, and dashed black lines show trailing 7-day moving averages. Columns represent focal taxa and rows represent monitoring stations. Note that y-axis scales differ among taxa to accommodate differences in activity magnitude.

The three focal taxa showed contrasting spatial and seasonal patterns (Figure 3). *Pipistrellus pipistrellus* was active throughout most of the monitoring period at all four stations. In contrast, *P. nathusii* activity was strongly concentrated in late summer and autumn: 84 to 93% of passes at individual stations occurred after 14 August, and the highest nightly counts fell within a narrow window between 26 and 29 September at all four stations. On these four nights, activity of *P. pipistrellus* was also elevated at three of the four stations (2.4–6.4 times the mean of the neighbouring nights). Autumn activity was greatest at ACF and ITU, although pronounced peaks were also recorded at Sarıyer Forest and Yıldız Park. Spring *P. nathusii* activity was low at all stations (1097 passes across 63 surveyed station-nights between 7 April and 31 May 2022), and no discrete spring peak was evident in the raw data.

The *N. noctula*/*N. leisleri* acoustic group was recorded less frequently and in more discrete seasonal pulses. Autumn accounted for more than 90% of this group’s activity at ACF, ITU, and Yıldız Park, whereas Sarıyer Forest also had a substantial spring component. The strongest activity occurred at ACF, where nightly counts peaked in late September. Eight of the 11 positive nights for *N. lasiopterus* occurred during September and October. Detections were concentrated in late September, when the species was recorded on five of 10 sampled nights between 16 and 30 September, coinciding with the peak of *P. nathusii* activity. Overall, the raw data indicate broad seasonal activity in *P. pipistrellus* and a marked autumn concentration in both migratory taxa, with spring activity of the migratory taxa weak and poorly resolved.

### 3.3. Site-Specific Seasonal Phenology

The full-season generalized additive models, including the calendar-night random effect, explained a substantial proportion of variation in hourly activity: 49.0% of deviance for *P. pipistrellus*, 66.5% for *P. nathusii*, and 66.2% for the *N. noctula*/*N. leisleri* acoustic group (Table S3). Models allowing site-specific seasonal patterns had substantially lower AIC than models with a common seasonal pattern (site-specific versus common seasonal smooths: *P. pipistrellus* ΔAIC = 274; *P. nathusii* ΔAIC = 235; the *N. noctula*/*N. leisleri* acoustic group ΔAIC = 196; Figure S1). Cross-validation supported the site-specific patterns for *P. nathusii* and the *N. noctula*/*N. leisleri* acoustic group, but not for *P. pipistrellus* (Table S3). Consistent with the raw data, *P. pipistrellus* showed relatively broad activity across the season at all stations; its fitted autumn maxima fell at the boundaries of the autumn window, whereas in spring the fitted curve at ACF reached an internal maximum in late April, just before the sampling interruption and with wide confidence intervals (Figure S1c). In contrast, the fitted autumn maxima of *P. nathusii* fell within the sampling period at all four stations (Sarıyer Forest, 14 September; Yıldız Park, 17 October; ITU, 26 October; ACF, 31 October), at ACF and ITU later than the raw nightly peaks of 26–29 September. The fitted autumn maxima of the *N. noctula*/*N. leisleri* acoustic group fell within the sampling period at ACF (18 September), ITU (18 October), and Sarıyer Forest (24 October); at Sarıyer Forest, this autumn peak was accompanied by the spring activity noted above. The highest fitted spring maximum of this group that did not coincide with the start of sampling, however, was at ITU in late April, exceeding that at Sarıyer Forest in early May (Figure S1a). Because this maximum falls immediately before the interruption in sampling at ITU (27 April–10 June) and is bounded by very wide confidence intervals, it is not interpreted as an estimated spring peak. All spring maxima of *P. nathusii* coincided with the start of site-specific sampling periods and were therefore treated as boundary maxima rather than estimated seasonal peaks. Because day of year and temperature were each strongly concurve with the remaining model terms (worst concurvity 0.89–0.90; Table S3), seasonal and temperature effects could not be fully separated, and the fitted seasonal smooths should be interpreted together with the temperature responses described below.

### 3.4. Within-Night Activity, Temperature, Moon Phase, and Precipitation

Time since sunset had a significant nonlinear effect for all taxa (all *p* < 0.001), with activity concentrated in the first hours after sunset and secondary activity later in the night (Figure S2; Table S4). Temperature was likewise associated with activity in all three groups (*P. pipistrellus*: EDF = 6.35; *P. nathusii*: EDF = 6.32; the *N. noctula*/*N. leisleri* acoustic group: EDF = 3.65; all *p* < 0.001). Predicted activity increased from low temperatures and peaked at approximately 19 to 23 °C before levelling off or declining at higher temperatures.

Moon phase and precipitation were retained as covariates (Table S4) but are not interpreted further, because lunar effects cannot be separated from cloud cover and seasonal timing within a single year and heavy-rainfall nights were excluded from sampling.

The complementary presence–absence models discriminated well between active and inactive hours (AUC = 0.911, 0.889, and 0.862 for *P. pipistrellus*, *P. nathusii*, and the *N. noctula*/*N. leisleri* acoustic group, respectively) and retained the same significant seasonal, temperature, and time-since-sunset terms as the count models. Other terms differed between the two model types. Moon phase for *P. pipistrellus* (*p* = 0.008), precipitation for *P. nathusii* (*p* < 0.001), and wind speed (*p* = 0.001) and precipitation (*p* = 0.004) for the *N. noctula*/*N. leisleri* acoustic group were significant only in the presence–absence models (Table S4).

### 3.5. Wind Speed and Wind Direction

Wind responses in the full-season models differed among taxa (Figure S3; Table S4). Wind speed was associated with activity of *P. pipistrellus* (EDF = 5.93) and *P. nathusii* (EDF = 5.11; both *p* < 0.001), but not with that of the *N. noctula*/*N. leisleri* acoustic group (EDF = 2.56, *p* = 0.17). Wind direction was associated with activity of *P. pipistrellus* (EDF = 2.36, *p* = 0.015) and the *N. noctula*/*N. leisleri* acoustic group (EDF = 2.12, *p* = 0.010), with the highest predicted activity of the latter under easterly winds, but not with that of *P. nathusii* (EDF = 3.40, *p* = 0.33).

### 3.6. Seasonal Wind Associations

Wind directions spanned nearly the full compass in both seasonal windows, with 22 of 24 directional sectors represented in spring and all 24 in autumn, but their frequency was uneven and dominated by northeasterly winds (Figure S4). Winds with a positive tailwind component along the expected movement axis occurred in 39.7% of spring hourly observations and 71.7% of autumn observations. Supportive winds were therefore available in relatively fewer spring hours but in most autumn hours.

Wind associations were examined separately within the two seasonal windows using the spring–autumn window subset of the data. Two complementary formulations were fitted for each focal taxon: a circular wind-direction smooth, either common to both seasons or allowed to differ by season, and a migration-relative tailwind component computed against the assumed spring (45°, NE) and autumn (225°, SW) movement axes (Table 1).

**Figure 4:**
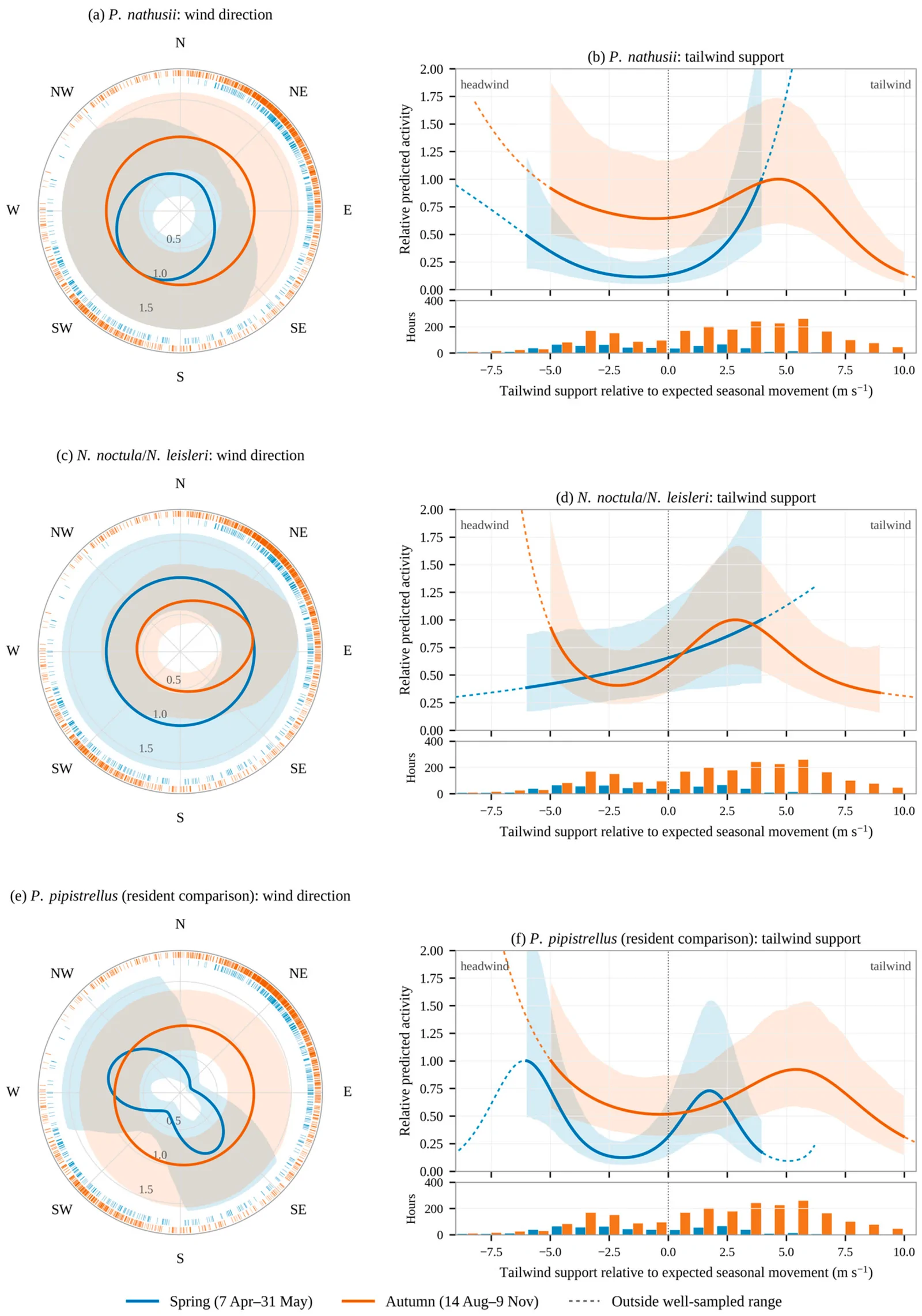
Season-specific associations between bat activity and wind during the spring (7 April–31 May) and autumn (14 August–9 November 2022) windows for *Pipistrellus nathusii* (**a**,**b**), the *Nyctalus noctula*/*N. leisleri* acoustic group (**c**,**d**), and the resident comparison species *P. pipistrellus* (**e**,**f**). (**a**,**c**,**e**) Predicted activity as a function of 10 m wind direction (direction from which the wind blows). (**b**,**d**,**f**) Predicted activity as a function of tailwind support relative to the assumed seasonal movement axis (toward 45° in spring and 225° in autumn); positive values indicate supporting winds. Predictions are from negative-binomial GAMs including a calendar-night random effect and AR(1) correction, averaged over stations at seasonal mean values of the other covariates with the random effect excluded, and scaled to the seasonal maximum within the well-sampled range. Shaded bands are 95% confidence intervals from 500 posterior simulations. Solid lines show the well-sampled range (≥30 hourly records and ≥5 h with activity per 1 m s^−1^ bin); dashed lines show extrapolation outside this range, which is not interpreted. Outer tick rings (**a**,**c**,**e**) show the wind direction of each hourly record, and bars below (**b**,**d**,**f**) give the number of hourly records per 1 m s^−1^ bin. Weather data: NASA POWER, converted from UTC to local time (UTC + 3).

After accounting for night-level variation, allowing wind responses to differ between the spring and autumn windows improved AIC only modestly for *P. nathusii* (ΔAIC = −12.2 for wind direction and −17.1 for tailwind support) and the *N. noctula*/*N. leisleri* acoustic group (ΔAIC = −5.0 and −9.2), and in neither taxon did it consistently improve cross-validated prediction (Table 1). The resident *P. pipistrellus* showed larger seasonal differences in AIC (ΔAIC = −43.4 and −62.7), which were likewise not consistently supported by cross-validation. In *P. nathusii* and *P. pipistrellus* alike, wind direction was significantly associated with activity in spring (*p* = 0.007 and *p* = 0.001; highest predicted activity under winds from approximately 215° and 287°, respectively) but not in autumn, whereas tailwind support was significantly associated with activity in both seasons (Table S4). Within the well-sampled range, the autumn tailwind responses of the two species had nearly the same shape (r = 0.82; Figure 4): activity was elevated under strong headwinds, lowest near neutral conditions, and highest under moderate tailwinds of approximately 4–5 m s^−1^, and declined under stronger tailwinds. In spring, *P. nathusii* activity was lowest near neutral wind support and increased towards the strongest well-sampled tailwinds (+4 m s^−1^).

For the *N. noctula*/*N. leisleri* acoustic group, wind direction was not associated with activity in spring, whereas autumn activity was highest under easterly winds (approximately 75°; *p* = 0.026). Tailwind support was not associated with spring activity but was strongly associated with autumn activity (*p* < 0.001), with the highest predicted activity under moderate tailwinds of approximately 3 m s^−1^ and lower activity under stronger tailwinds (Figure 4; Table S4).

## 4. Discussion

This study provides the first multi-site, model-based acoustic assessment of seasonal bat activity along the Bosphorus. Three findings stand out. First, the two migratory taxa showed strongly autumn-concentrated activity, with *P. nathusii* peaking synchronously at all four stations within a four-night window in late September, whereas *P. pipistrellus* remained active across the season. Second, site-specific seasonal patterns were supported for the migratory taxa, whose fitted autumn maxima fell within the sampling period at all (*P. nathusii*) or three of the four stations (*N. noctula*/*N. leisleri*). Third, once night-level variation was accounted for, wind associations of the migratory taxa differed only weakly between spring and autumn, and the resident *P. pipistrellus* showed equal or larger seasonal differences. Taken together, the seasonal phenology is consistent with migratory movement through the Istanbul–Bosphorus region, whereas the wind associations reflect weather effects on local activity rather than providing independent evidence of directed movement.

### 4.1. Seasonal Phenology and Evidence of Migration

The autumn concentration of *P. nathusii* activity, with 84 to 93% of passes recorded after mid-August and peak counts between 26 and 29 September, matches the timing of autumn passage reported along the Baltic and North Sea coasts [11,12,13,14] and at high-elevation Alpine passes [52]. Although *P. nathusii* and *P. kuhlii* cannot always be distinguished reliably from echolocation calls alone, and some contribution of *P. kuhlii* cannot be excluded, the strong autumn concentration is consistent with this acoustic category being dominated by the migratory *P. nathusii*. This timing is also consistent with the September to October *P. nathusii* peak reported from the northern Marmara coast in 2020, where activity remained elevated throughout September and reached its highest single-night value in early September [34]. The near-simultaneous peak at all four Istanbul stations is notable: the stations span approximately 17 km along the Bosphorus and contrasting urban and forested settings, making such synchrony difficult to explain by site-specific local activity alone. However, because three of the four stations share the same weather series, synchrony is also expected if activity at all sites responded to the same favourable nights. Indeed, *P. pipistrellus* activity was also elevated on these nights at three of the four stations, so the synchronous peak probably reflects nights that were favourable for bat activity in general and cannot by itself indicate passage. Synchronous pulses would nevertheless also be expected during migratory passage, when movements may become concentrated on a limited number of nights under favourable conditions [18,19]. The evidence for migration therefore rests on the seasonal concentration of activity in the migratory taxa, which the largely resident *P. pipistrellus* does not show. The autumn increase may nevertheless reflect arrival at mating or wintering sites as well as transit; because monitoring ended in early November, passage and arrival cannot be distinguished with our data.

Activity of the *N. noctula/N. leisleri* acoustic group showed a similar but more discrete autumn pulse, with more than 90% of passes at three of the four stations recorded during autumn. This pattern is consistent with the well-documented autumn migration of *N. noctula* and *N. leisleri* along a northeast-to southwest axis [10,51], the predominantly autumnal occurrence of *N. noctula* reported from the Marmara coast [34], and the prominence of these species among turbine fatalities along the Black Sea coast [8]. In contrast, Sarıyer Forest showed a more substantial spring component. Given that this was the northernmost and most forested site, this pattern may reflect spring passage, local roosting activity, or both, and cannot be distinguished from acoustic detections alone.

Spring migration was less clearly resolved from seasonal phenology. *Pipistrellus nathusii* activity was comparatively low in spring, and several apparent maxima occurred at the beginning of the sampling period and were therefore treated as boundary effects. Consequently, the acoustic time series alone does not provide clear evidence for a discrete spring passage. Such seasonal asymmetry has been reported in acoustic migration studies, where spring movements are often less temporally concentrated and less conspicuous than autumn passage [11]. It may also reflect partial migration, which is well documented in European migratory bats [51], such that some individuals detected during spring and summer may represent locally active rather than transient bats. In addition, *P. nathusii* was detected on the first sampling night, and several maxima occurred near the start of sampling, suggesting that spring passage may have begun before monitoring started; this is consistent with the April peak of *P. nathusii* fatalities reported from wind farms in Thrace [46]. The weak spring signal may also reflect the behaviour of spring migrants, which typically move quickly towards their summer ranges and spend little time at intermediate sites.

### 4.2. Spatial Variation and the Urban Context

Beyond these temporal patterns, activity and species composition varied substantially among stations. Atatürk City Forest, located within an urban-to-natural transition zone, supported the highest species richness, the strongest activity of the *N. noctula/N. leisleri* acoustic group, and 10 of the 11 positive nights of the globally Vulnerable *Nyctalus lasiopterus*, whereas Yıldız Park, the most heavily urbanized site, supported the fewest species. ACF and Sarıyer Forest also supported the Near Threatened *R. euryale* [53], whereas all remaining species identified to species level are listed as Least Concern. This pattern is consistent with studies from other Mediterranean metropolitan areas showing reduced bat richness and activity with increasing urbanization, while large urban green spaces can retain important habitat for bats [54]. Artificial light at night may also contribute to lower richness at more urban sites by altering the activity of *Pipistrellus* and deterring light-sensitive taxa [6]. Nevertheless, migratory taxa were detected at all four stations, and the autumn *P. nathusii* peak was evident throughout the network, although activity levels differed markedly among sites. This suggests that seasonal movement extends across the urban landscape, while also leaving open the possibility that bats preferentially use particular crossing points or green-space connections along the Bosphorus. At ACF, however, the *P. nathusii* acoustic category was also recorded regularly in summer (June to mid-August: mean 204 passes per night on 11 sampled nights, with up to 612 passes on 30 June), in contrast to the other three stations (mean 9–67 passes per night). This summer activity cannot be explained by passage and may indicate a locally summering group of *P. nathusii* (e.g., males or non-migratory individuals), a contribution of *P. kuhlii* to this acoustic category, or both. Several species recorded acoustically at wind farms in Thrace [46], such as *Miniopterus schreibersii*, *P. pygmaeus*, *Hypsugo savii*, and *Vespertilio murinus*, were not identified in our recordings. This may partly reflect differences in habitat, as our stations were located in urban parks and peri-urban forests rather than open rural landscapes, and the absence of suitable roosts such as caves for *M. schreibersii* or rocky cliffs for *H. savii* near the stations.

The repeated detection of *N. lasiopterus* at Atatürk City Forest deserves particular attention. The greater noctule is globally classified as Vulnerable, reflecting population decline and fragmentation [55], and has been recorded from several localities in Türkiye [56]. Recent GPS tracking has demonstrated long-distance movements exceeding 2500 km, confirming its capacity for extensive seasonal migration [57]. The species is also unusual in regularly preying on nocturnally migrating passerines in flight [58], which is particularly relevant given the large numbers of migratory birds concentrated around the Bosphorus. In our data, detections at Atatürk City Forest were clustered in late September, when the species was recorded on half of the sampled nights during the peak of *P. nathusii* activity, and most passes occurred shortly after sunset. Whether *N. lasiopterus* uses the region primarily as a migration corridor, a seasonal foraging area, or both remains unknown, but its repeated occurrence adds a globally threatened species to the conservation significance of the Istanbul–Bosphorus landscape.

Site-specific seasonal smooths were better supported than a common seasonal pattern for the two migratory taxa, also in cross-validation, indicating that phenology varied among stations even within a single metropolitan area. This fine-scale heterogeneity may reflect differences in habitat structure, position along the Bosphorus, and local exposure to prevailing winds, and highlights the value of multi-site monitoring for resolving migration patterns within potential corridors.

### 4.3. Wind Associations

We also asked whether wind associations of migratory bats differed between spring and autumn in a manner consistent with seasonally reversed migration. Wind directions covered nearly the full compass in both periods, but their frequency was strongly uneven and dominated by northeasterly winds. Consequently, winds providing positive tailwind support along the expected migration axis occurred during only 39.7% of spring hours but 71.7% of autumn hours. Supportive winds were therefore relatively uncommon in spring but prevalent in autumn, an asymmetry that is important for interpreting the seasonal responses.

Once night-level variation was accounted for, the wind associations showed no such seasonal contrast. Season-specific wind responses of *P. nathusii* and the *N. noctula*/*N. leisleri* acoustic group improved AIC only modestly and did not consistently improve cross-validated prediction, and the resident *P. pipistrellus* showed equal or larger seasonal differences. In *P. nathusii* and *P. pipistrellus* alike, wind direction was associated with activity in spring but not in autumn, and the autumn tailwind responses of the two species had nearly the same shape. Our data therefore provide no evidence for the wind-assisted spring migration reported for other European bats [18,19] and offshore *P. nathusii* passage [13]. However, these studies concerned bats in active passage, often over open water, whereas our detectors were located in sheltered forest, park, and campus habitats, where bats need not contend with the wind to the same extent.

Ground-based acoustic detectors measure local activity rather than passage itself, and migrants may nevertheless have contributed to the elevated autumn activity under headwinds. Bats travelling rapidly under supportive winds may spend relatively little time within detector range, whereas individuals encountering opposing winds may slow, descend, forage, or remain temporarily within an area, thereby generating more detections. *Pipistrellus nathusii* is known to combine migration with opportunistic feeding along its route in a fly-and-forage strategy [59], making such behaviour plausible. In sheltered sites, bats may also forage or wait during unfavourable weather. Moreover, autumn coincides with the mating period, when migrants are under less time pressure than in spring and may remain longer in suitable areas [43]. Both processes would increase hourly activity independently of passage.

Migratory movement itself is also unlikely to be strictly unidirectional. Three-dimensional acoustic tracking at a major Baltic corridor showed that although most *P. nathusii* moved in the expected direction, a substantial minority flew antiparallel, particularly under stronger winds [26]. Such reverse or cross-axis movements would weaken any simple relationship between local acoustic activity and a single migration axis. In addition, the decline in activity at the strongest supportive winds may reflect the generally negative effect of high wind speeds on bat flight rather than avoidance of tailwind support itself [13,60].

A weather-related explanation is more parsimonious. Wind conditions in Istanbul are dominated by two contrasting regimes: cool north-easterly winds and warm south-westerly winds (Figure S4). Because the assumed migration axis reverses between seasons, a season-invariant preference for warm south-westerly airflow would appear as a tailwind response in spring and as a headwind response in autumn. In spring, *P. nathusii* activity was highest under south-westerly winds (approximately 215°), whereas no directional association was detected in autumn, and the resident *P. pipistrellus* showed the same seasonal pattern. Part of the spring association may therefore reflect the warmer conditions and higher insect availability associated with south-westerly airflow. The wind associations of the resident *P. pipistrellus* are most parsimoniously explained by these warmer conditions and by local sheltering effects at the detector sites, rather than by migratory movement. The distinct seasonal phenology of *P. nathusii* and the *N. noctula*/*N. leisleri* acoustic group, in contrast to *P. pipistrellus*, therefore remains the main evidence for migration in our data, whereas the wind associations are best regarded as describing how local activity at sheltered sites varied with weather.

### 4.4. Temperature, Within-Night Timing, Moon Phase, and Precipitation

In addition to seasonal phenology, temperature and time since sunset were the most consistent predictors across taxa. Predicted activity increased with temperature and was highest at approximately 19 to 23 °C before declining, broadly consistent with patterns reported for temperate insectivorous bats and migratory activity at wind-energy sites [13,60]. This range also overlaps the 18.8 to 21.2 °C conditions associated with peak September *P. nathusii* activity on the Marmara coast [34]. Activity was concentrated in the first hours after sunset, with secondary increases later in the night, consistent with the strong nocturnal timing of both local foraging and migratory activity.

Moon phase and precipitation were associated with activity of the migratory taxa, but we do not interpret these effects. Moonlight depends on both lunar phase and cloud cover, which was not measured, and within a single year lunar phase is confounded with seasonal timing; because the date of full moon during the autumn migration period shifts by several weeks between years, lunar effects can only be assessed with multi-year data. Precipitation effects were estimated over a restricted range of conditions because heavy-rainfall nights were excluded from sampling.

### 4.5. Limitations and Future Directions

Several limitations qualify our interpretation. The study covers a single year and four stations, all located on the European side of the Bosphorus, and therefore cannot resolve interannual variability or determine whether bats preferentially cross at particular sections of the strait. Spring coverage at ITU and ACF was also interrupted by a detector failure, limiting our ability to resolve a distinct spring phenological peak at these two stations. Meteorological variables were obtained from NASA POWER reanalysis at 10 m height and may not fully represent conditions within sheltered urban and forest habitats or the local wind fields generated by the Bosphorus. Wind conditions experienced by bats at flight heights may also differ substantially from those represented at 10 m. Three of the four stations share identical NASA POWER weather series, so observations from the same night are not independent across these stations; the calendar-night random effect accounts for conditions shared among stations, and the hourly weather data were aligned to local civil time (UTC + 3) before matching (Section 2.2). Day of year and temperature were also strongly concurve with the other model terms, so seasonal and temperature effects cannot be fully separated. Residual dispersion was close to one in most count models (Pearson dispersion 1.19–1.34) but remained moderately elevated in the season-specific models for the *N. noctula*/*N. leisleri* acoustic group (1.43–1.56; Table S3), so standard errors for this group in the seasonal wind analyses may be somewhat underestimated. In addition, the first and last hourly intervals of each night were incomplete, because recording began 30 min before sunset and ended 30 min after sunrise; recording duration was not included as an offset, so activity in these intervals may be slightly underestimated, which mainly affects the within-night activity profile captured by the smooth of hours after sunset. Wind noise may reduce detectability at high wind speeds and could contribute to the lower activity recorded under the strongest winds. Finally, the *P. nathusii* acoustic category may include *P. kuhlii*, particularly at the more urban stations and in spring, when *P. nathusii* activity was low; spring results and between-station comparisons for this taxon should therefore be interpreted with caution.

Wind availability was strongly uneven, with northeasterly winds dominating both seasonal windows. Although almost all wind directions were represented, some portions of the fitted responses, particularly strong autumn headwinds, were supported by relatively few observations and should therefore be interpreted cautiously. The migration axes used to calculate tailwind support were based on expected seasonal movement rather than directly observed flight directions, and this limitation is particularly relevant for the *N. noctula/N. leisleri* acoustic group. More fundamentally, acoustic detectors measure local activity rather than movement direction. They cannot distinguish bats passing rapidly through a site from individuals foraging, stopping over, or making local movements, which limits the behavioural interpretation of the observed wind responses. This limitation is accentuated by the sheltered location of our detectors in forest, park, and campus habitats. Given the short detection range of *P. nathusii* (approximately 20–25 m [42]), bats flying higher and exposed to the full effect of the wind were probably rarely recorded, except for the louder *Nyctalus* species, so the wind associations reported here describe local activity rather than the behaviour of bats in passage. Moreover, no reference sites away from the strait were sampled, so our design cannot show that bats concentrate along the Bosphorus or cross it. Unlike soaring birds, bats readily fly over open water, as shown by records of *P. nathusii* over the western Black Sea [33], and the similar autumn peak on the northern Marmara coast [34] is equally consistent with broad-front movement. The role of the Bosphorus as a migration corridor should therefore be regarded as a hypothesis to be tested.

Future studies should address these uncertainties through multi-year monitoring on both sides of the Bosphorus and at additional stations along the Black Sea coast and Dardanelles. Such a design would test whether the late-September activity peak is consistent among years and whether migration is concentrated at particular crossing points rather than distributed broadly across the region. Local meteorological measurements, ideally including wind at biologically relevant flight heights, would improve characterization of the conditions actually experienced by migrating bats. Most importantly, approaches capable of resolving flight direction, including acoustic tracking arrays, radar, and biologging, would allow direct separation of migratory passage from local foraging and stopover activity and test whether migrating bats select particular wind conditions for passage.

### 4.6. Conservation Implications

Despite these limitations, the results have practical implications for conservation and wind-energy planning. Long-distance migratory bats are disproportionately represented among wind-turbine fatalities [7], and substantial mortality of *P. nathusii* and *N. noctula*, concentrated in July and August, has already been documented at wind farms along the western Black Sea coast [8]. Our results indicate that migratory bat activity in Istanbul becomes strongly concentrated during late September and occurs across both forested and highly urbanized parts of the city. Activity levels nevertheless differed among stations, leaving open the possibility that particular green-space networks or sections of the Bosphorus are preferentially used during migration. Identifying such spatial concentrations should therefore be an important component of future wind-energy and infrastructure planning in the wider Thrace region.

The strong seasonal and meteorological structure of activity also has implications for operational mitigation. Curtailment is most effective when it can be targeted to periods of elevated collision risk rather than applied uniformly throughout the season [21]. The concentration of migratory activity within a relatively narrow autumn period, together with its association with temperature and wind conditions, suggests that locally calibrated activity and weather thresholds could help identify periods when turbine operation poses the greatest risk. Site-specific monitoring, following the EUROBATS guidelines [21], would nevertheless be required before translating the relationships observed here into operational curtailment rules.

The conservation relevance of migratory bats in the Istanbul–Bosphorus region extends beyond their global threat categories. Although *P. nathusii*, *N. noctula*, and *N. leisleri* are globally classified as Least Concern, long-distance migration exposes individuals to cumulative pressures across multiple countries and landscapes. The repeated detection of the globally Vulnerable *N. lasiopterus*, particularly at Atatürk City Forest, further emphasizes the potential importance of the Istanbul–Bosphorus landscape for migratory bats of conservation concern. Because individuals moving through the region may connect populations across southeastern Europe, the Black Sea region, and Asia, protection of migration corridors and stopover habitats requires a transboundary perspective consistent with the objectives of EUROBATS.

Together, our results suggest that conservation planning around the Bosphorus should consider both where migratory bats concentrate and when and under which atmospheric conditions seasonal movements occur. Combining spatial identification of potential crossing and stopover areas with temporally targeted mitigation could provide a practical framework for reducing risks to migratory bats while accommodating continued infrastructure and wind-energy development in the region.

## 5. Conclusions

Passive acoustic monitoring at four sites along the Bosphorus revealed strongly autumn-concentrated and site-synchronous activity in *P. nathusii* and the *N. noctula/N. leisleri* acoustic group, contrasting with the broader seasonal activity of *P. pipistrellus*. Wind associations were similar in migratory and resident species and therefore do not provide independent evidence of directed movement. These patterns are consistent with the Istanbul–Bosphorus region being used by migrating bats and support, but do not yet demonstrate, the hypothesis that the Bosphorus functions as a corridor for seasonal bat movement between Europe and Asia. The repeated detection of the globally Vulnerable *N. lasiopterus* further increases the conservation significance of the area. Given the concentration of migratory activity within a relatively narrow seasonal window, the Bosphorus and wider Marmara region should receive greater attention in migration monitoring, spatial planning, and targeted mitigation associated with wind-energy development.

## Figures and Tables

**Table 1:** Comparison of common and season-specific wind responses within the spring–autumn window subset (negative-binomial GAMs with a calendar-night random effect; corrected weather data). ΔAIC, ΔCV RMSE, and ΔCV MAE are differences between the season-specific and the common model; negative ΔAIC favours the season-specific model, and positive ΔCV RMSE or ΔCV MAE indicates poorer cross-validated prediction by the season-specific model. Cross-validation was five-fold, grouped by calendar night.

Taxon	Wind Term	ΔAIC	Deviance Explained (Common/Seasonal)	ΔCV RMSE	ΔCV MAE
*P. nathusii*	Circular direction	−12.2	64.9%/65.3%	+0.64	+0.13
*P. nathusii*	Migration-relative tailwind	−17.1	65.1%/65.6%	+0.17	−0.05
*N. noctula/N. leisleri*	Circular direction	−5.0	58.2%/58.5%	+0.03	+0.00
*N. noctula/N. leisleri*	Migration-relative tailwind	−9.2	59.3%/59.9%	−0.39	+0.08
*P. pipistrellus*	Circular direction	−43.4	46.4%/47.5%	+37.7	+2.94
*P. pipistrellus*	Migration-relative tailwind	−62.7	46.0%/47.3%	+4.12	−0.18

## Data Availability

The data presented in this study are available on request from the corresponding author. The R code used for data processing and analysis is provided as an R Markdown document in Supplementary File S1.

## Supplementary Materials

The following supporting information can be downloaded at https://www.mdpi.com/article/10.3390/ani16213346/s1. Table S1: Passive acoustic monitoring stations in Istanbul, Türkiye, April–November 2022; Table S2: Bat species and acoustic groups recorded at the four monitoring stations between April and November 2022.; Table S3: Overview and diagnostics of the generalized additive models (GAMs) fitted to hourly bat activity at four monitoring stations in Istanbul, April–November 2022; Table S4: Approximate significance of smooth terms in the GAMs summarized in Table S3; Figure S1: Site-specific seasonal activity of (a) the *Nyctalus noctula*/*N. leisleri* acoustic group, (b) *Pipistrellus nathusii* and (c) *P. pipistrellus* at the four monitoring stations; Figure S2: Partial effects of air temperature (left column) and hours after sunset (right column) on predicted hourly activity of *P. pipistrellus* (top), *P. nathusii* (middle) and the *N. noctula*/*N. leisleri* acoustic group (bottom) from the full-season negative-binomial GAMs with a calendar-night random effect; Figure S3: Partial effects of 10 m wind direction (left; degrees, meteorological convention, i.e. the direction from which the wind blows, modelled as a cyclic smooth) and 10 m wind speed (right; m s⁻¹) on predicted hourly activity of the *N. noctula*/*N. leisleri* acoustic group (top), *P. nathusii* (middle) and *P. pipistrellus* (bottom) from the full-season GAMs with a calendar-night random effect; Figure S4: Wind conditions during the spring (7 April–31 May) and autumn (14 August–9 November 2022) windows; Figure S5: Nightly detections of less frequently recorded bat taxa at the four monitoring stations, April–November 2022; Figure S6: Example spectrograms of search-phase echolocation sequences of (a) *Nyctalus noctula* (Atatürk City Forest, 19 October 2022, 18:21; 36 calls), (b) *N. lasiopterus* (ITU Campus, 23 September 2022, 23:56; 10 calls), and (c) *Tadarida teniotis* (Atatürk City Forest, 25 October 2022, 20:54; 8 calls), illustrating the identification criteria described in Section 2.3 of the main text; File S1: R Markdown document containing the R code used for data processing, statistical modelling, and figure generation.
