# Supplementary material for: Seasonal Dynamics of Bat Activity in the Bosphorus Region (Istanbul, Türkiye): Acoustic Evidence for a Potential Migration Corridor Between Europe and Asia
Source: Animals (Basel). 2026 Oct 23;16(21):3346. doi: 10.3390/ani16213346 (PMC13762421; doi:10.3390/ani16213346)
Supplement: Supplementary file 1 [file animals-16-03346-s001.zip › animals-4584487-supplementary/Seasonal Dynamics of Bat Activity in the Bosphorus Region_Supplementary Materials_V7.pdf]

Supplementary Materials

Seasonal Dynamics of Bat Activity in the Bosphorus Region (Istanbul, Türkiye): Acoustic Evidence for a Potential Migration Corridor Between Europe and Asia

Tuğçe Nur İlbaş Gür and Emrah Çoraman *

[1] Eurasia Institute of Earth Sciences, Istanbul Technical University, 34467, Istanbul, Türkiye

ilbas15@itu.edu.tr

* Correspondence: coraman@itu.edu.tr

Table S1. Passive acoustic monitoring stations in Istanbul, Türkiye, April–November 2022. Coordinates are in decimal degrees (WGS 84). Distance to shoreline is the straight-line distance from the station to the shoreline of the Bosphorus. Start and end dates give the first and last recording nights at each station (each night is dated by the date of sunset, so that recordings made after midnight belong to the preceding date); "Nights" is the number of processed station-nights included in the analytical dataset, and "Total hours" is the number of hourly observations included in the analytical dataset.

Station	Latitude	Longitude	Habitat description	Distance to Bosphorus shoreline (km)	Start date	End date	Nights	Total hours
Sarıyer Forest	41.19631	29.0695	Forested peri-urban site in the northern forest belt.	1.3	20/4/2022	10/11/2022	105	1031
Atatürk City Forest (ACF)	41.13184	29.03393	Urban forest with mixed woodland and recreational clearings.	2.0	7/4/2022	9/11/2022	72	913
ITU Ayazağa Campus (ITU)	41.10408	29.03148	University campus with mixed built, wooded and open green areas.	1.9	7/4/2022	4/11/2022	64	742
Yıldız Park	41.04564	29.01419	Historic urban park with mature trees and landscaped green space in a densely urbanized area.	0.4	7/4/2022	3/11/2022	74	927

Table S2. Bat species and acoustic groups recorded at the four monitoring stations between April and November 2022. Total passes is the number of bat passes assigned to each taxon; first and last positive night are the first and last recording nights with at least one pass. Acoustic groups (e.g. *N. noctula*/*N. leisleri*) contain passes that could not be reliably assigned to a single species. *Plecotus auritus* and *P. austriacus* cannot be separated reliably from echolocation calls and are reported as *Plecotus* sp. Station abbreviations follow Table S1.

Species / acoustic group	Total passes	Stations detected	First positive night	Last positive night
Rhinolophus euryale	8	ACF; Sarıyer Forest	10/6/2022	26/10/2022
R. hipposideros	7	ACF; Sarıyer Forest; Yıldız Park	1/5/2022	19/10/2022
R. ferrumequinum	107	ACF; ITU; Sarıyer Forest	23/4/2022	12/9/2022
R. euryale/*R. mehelyi*/*R. hipposideros*	15	ACF; Sarıyer Forest; Yıldız Park	8/6/2022	26/10/2022
R. mehelyi/*R. hipposideros*	7	Sarıyer Forest; Yıldız Park	19/9/2022	9/10/2022
Plecotus sp.	27	ACF; ITU; Sarıyer Forest	17/5/2022	7/11/2022
Myotis spp.	329	ACF; ITU; Sarıyer Forest; Yıldız Park	10/4/2022	4/11/2022
Pipistrellus pipistrellus	212,012	ACF; ITU; Sarıyer Forest; Yıldız Park	7/4/2022	10/11/2022
P. nathusii	40,647	ACF; ITU; Sarıyer Forest; Yıldız Park	7/4/2022	9/11/2022
Eptesicus serotinus	70	ACF; ITU; Sarıyer Forest; Yıldız Park	27/4/2022	25/10/2022
Nyctalus noctula	421	ACF; ITU; Sarıyer Forest; Yıldız Park	7/4/2022	4/11/2022
N. leisleri	341	ACF; ITU; Sarıyer Forest; Yıldız Park	7/4/2022	4/11/2022
N. lasiopterus	22	ACF; ITU	10/4/2022	23/10/2022
N. noctula/*N. leisleri*	5,606	ACF; ITU; Sarıyer Forest; Yıldız Park	7/4/2022	10/11/2022
N. noctula/*N. leisleri*/*E. serotinus*	28	ACF; ITU; Sarıyer Forest; Yıldız Park	23/4/2022	29/9/2022
Tadarida teniotis	7	ACF; ITU	14/9/2022	30/10/2022

Table S3. Overview and diagnostics of the generalized additive models (GAMs) fitted to hourly bat activity at four monitoring stations in Istanbul, April–November 2022 (corrected NASA POWER weather data, UTC+3). All models were fitted with mgcv::bam (v1.9) with a first-order autoregressive (AR(1)) working correlation for within-night residual autocorrelation (ϱ). All count models include a random intercept for calendar night; its standard deviation is given. Full-season count models with site-specific seasonal smooths are the primary analyses; the models with a common seasonal smooth are the alternatives used in the model comparison in Section 3.3, and the presence/absence models are sensitivity analyses. The spring–autumn window models (7 April–31 May and 14 August–9 November 2022) are the season-specific formulations summarized in Table 1; their common-response alternatives are given there. Deviance explained is the proportion of null deviance accounted for by the model. Pearson dispersion is the ratio of the Pearson chi-square statistic to residual degrees of freedom. CV RMSE and CV MAE are the root mean squared error and mean absolute error of five-fold cross-validation with folds grouped by calendar night. Worst concurvity is the mgcv "worst" index (the degree to which a term can be reproduced by all other model terms) for the seasonal (day of year; maximum across stations) and temperature smooths; it was computed on the equivalent models without the calendar-night random effect, because a random intercept for night can reproduce any function of day of year and makes the index uninformative (= 1). The pairwise concurvity between day of year and temperature did not exceed 0.30 and that between day of year and moon phase did not exceed 0.11; the precipitation smooth had a worst index of 0.95 against the parametric (site) terms because precipitation was zero in most hours. AUC (area under the ROC curve) is reported for binomial models only. Smooth-term results are given in Table S4.

Taxon	Analysis	Response family	Hours	Station-nights	Deviance explained (%)	AIC	AR(1) ϱ	Pearson dispersion	Night random-effect SD	CV RMSE	CV MAE	Worst concurvity (day of year; temperature)	AUC
Pipistrellus pipistrellus	Full season, hourly counts, site-specific seasonal smooths (primary)	NB (θ = 0.54)	3,613	315	49.0	29,442.1	0.208	1.19	0.68	145.0	58.8	0.89; 0.90	–
	Full season, hourly counts, common seasonal smooth	NB (θ = 0.50)	3,613	315	44.8	29,715.6	0.210	1.26	0.70	93.2	51.5	–	–
	Full season, presence/absence (sensitivity; no night random effect)	Binomial	3,613	315	42.0	2,530.3	0.024	0.77	–	–	–	0.89; 0.90	0.911
	Spring–autumn windows, season-specific circular wind direction	NB (θ = 0.46)	2,867	240	47.5	21,403.6	0.208	1.23	0.88	138.6	47.5	0.74; 0.80	–
	Spring–autumn windows, season-specific tailwind/crosswind	NB (θ = 0.46)	2,867	240	47.3	21,403.1	0.209	1.25	0.89	110.8	46.8	0.75; 0.81	–
Pipistrellus nathusii	Full season, hourly counts, site-specific seasonal smooths (primary)	NB (θ = 0.72)	3,613	315	66.5	16,597.8	0.070	1.26	0.88	28.9	10.7	0.89; 0.90	–
	Full season, hourly counts, common seasonal smooth	NB (θ = 0.63)	3,613	315	63.0	16,832.9	0.074	1.30	0.95	29.8	11.1	–	–
	Full season, presence/absence (sensitivity; no night random effect)	Binomial	3,613	315	39.7	3,116.7	-0.005	0.93	–	–	–	0.89; 0.90	0.889
	Spring–autumn windows, season-specific circular wind direction	NB (θ = 0.73)	2,867	240	65.3	14,203.8	0.113	1.26	1.02	31.1	13.3	0.74; 0.80	–
	Spring–autumn windows, season-specific tailwind/crosswind	NB (θ = 0.74)	2,867	240	65.6	14,186.3	0.108	1.26	1.02	31.8	13.3	0.75; 0.81	–

Taxon	Analysis	Response family	Hours	Station-nights	Deviance explained (%)	AIC	AR(1) ϱ	Pearson dispersion	Night random-effect SD	CV RMSE	CV MAE	Worst concurvity (day of year; temperature)	AUC
N. noctula/*N. leisleri*	Full season, hourly counts, site-specific seasonal smooths (primary)	NB (θ = 0.57)	3,613	315	66.2	7,343.0	0.099	1.24	0.61	7.7	1.8	0.89; 0.90	–
	Full season, hourly counts, common seasonal smooth	NB (θ = 0.46)	3,613	315	61.2	7,539.2	0.120	1.34	0.69	7.9	1.8	–	–
	Full season, presence/absence (sensitivity; no night random effect)	Binomial	3,613	315	32.2	3,014.5	0.090	1.01	–	–	–	0.89; 0.90	0.862
	Spring–autumn windows, season-specific circular wind direction	NB (θ = 0.48)	2,867	240	58.5	6,958.3	0.114	1.43	0.73	8.9	2.3	0.74; 0.80	–
	Spring–autumn windows, season-specific tailwind/crosswind	NB (θ = 0.51)	2,867	240	59.9	6,922.8	0.100	1.56	0.75	8.5	2.4	0.75; 0.81	–

Table S4. Approximate significance of smooth terms in the GAMs summarized in Table S3 (all count models include a random intercept for calendar night). EDF, effective degrees of freedom; chi-square statistics and p-values are as reported by mgcv summary(). Day of year was fitted as a site-specific smooth in the full-season models (factor-smooth interaction). Wind direction and moon phase were fitted as cyclic smooths. In the season-specific models, separate smooths were fitted for the spring (7 April–31 May) and autumn (14 August–9 November 2022) windows; tailwind and crosswind components are defined relative to a northeastward (45°) axis in spring and a southwestward (225°) axis in autumn, so positive tailwind values indicate wind support in the expected seasonal direction. Site was included as a parametric factor in all models. EDF values near zero indicate terms shrunk to the null by penalization. Wind effects are nonlinear; significance should not be read as a simple positive or negative response (see Figures S3 and 4 and the main text).

Smooth term	EDF	Chi-square	p
Pipistrellus pipistrellus **— Full season, hourly counts (negative binomial, calendar-night random effect)**			
Day of year × Sarıyer Forest	1.00	7.57	0.006
Day of year × Atatürk City Forest	6.65	17.62	<0.001
Day of year × ITU Campus	1.91	52.98	<0.001
Day of year × Yıldız Park	1.00	4.54	0.033
Hours after sunset	6.94	144.19	<0.001
Temperature	6.35	72.99	<0.001
Wind speed	5.93	7.66	<0.001
Wind direction (cyclic)	2.36	10.31	0.015
Moon phase (cyclic)	<0.01	<0.01	0.331
Precipitation	1.00	9.74	0.002
Calendar night (random intercept)	110.14	2.54	<0.001
Pipistrellus pipistrellus **— Full season, presence/absence (binomial; sensitivity)**			
Day of year × Sarıyer Forest	3.56	25.58	<0.001
Day of year × Atatürk City Forest	6.97	91.15	<0.001
Day of year × ITU Campus	1.00	18.47	<0.001
Day of year × Yıldız Park	7.88	50.02	<0.001
Hours after sunset	6.84	349.95	<0.001
Temperature	5.29	322.62	<0.001
Wind speed	5.24	47.05	<0.001
Wind direction (cyclic)	3.35	18.77	<0.001
Moon phase (cyclic)	2.01	7.83	0.008
Precipitation	2.15	15.47	0.001
Pipistrellus pipistrellus **— Season-specific circular wind-direction model (spring and autumn windows)**			
Day within seasonal window	2.81	3.29	0.038
Hours after sunset	6.92	93.45	<0.001
Temperature	6.06	37.63	<0.001
Wind speed × Spring	4.87	4.84	<0.001
Wind speed × Autumn	5.98	8.70	<0.001
Wind direction (cyclic) × Spring	4.59	15.48	0.001
Wind direction (cyclic) × Autumn	0.64	0.57	0.261
Moon phase (cyclic)	<0.01	<0.01	<0.001
Precipitation	2.71	6.50	<0.001

Smooth term	EDF	Chi-square	p
Calendar night (random intercept)	82.37	3.48	<0.001
Pipistrellus pipistrellus **— Season-specific migration-relative tailwind/crosswind model (spring and autumn windows)**			
Day within seasonal window	2.95	3.90	0.007
Hours after sunset	6.92	92.05	<0.001
Temperature	5.78	40.57	<0.001
Tailwind component × Spring	4.82	8.17	<0.001
Tailwind component × Autumn	4.14	5.85	<0.001
Crosswind component × Spring	4.01	11.15	<0.001
Crosswind component × Autumn	1.00	2.40	0.122
Moon phase (cyclic)	<0.01	<0.01	0.036
Precipitation	2.63	7.51	<0.001
Calendar night (random intercept)	82.07	3.51	<0.001
Pipistrellus nathusii **— Full season, hourly counts (negative binomial, calendar-night random effect)**			
Day of year × Sarıyer Forest	7.03	33.29	<0.001
Day of year × Atatürk City Forest	7.51	7.86	<0.001
Day of year × ITU Campus	5.86	12.04	<0.001
Day of year × Yıldız Park	7.20	16.02	<0.001
Hours after sunset	6.89	194.16	<0.001
Temperature	6.32	52.97	<0.001
Wind speed	5.11	6.13	<0.001
Wind direction (cyclic)	3.40	5.96	0.329
Moon phase (cyclic)	1.29	9.86	0.189
Precipitation	1.00	1.72	0.190
Calendar night (random intercept)	114.70	4.29	<0.001
Pipistrellus nathusii **— Full season, presence/absence (binomial; sensitivity)**			
Day of year × Sarıyer Forest	6.73	190.37	<0.001
Day of year × Atatürk City Forest	7.32	92.85	<0.001
Day of year × ITU Campus	6.16	142.34	<0.001
Day of year × Yıldız Park	7.87	175.10	<0.001
Hours after sunset	6.75	624.47	<0.001
Temperature	6.24	169.50	<0.001
Wind speed	4.99	30.09	<0.001
Wind direction (cyclic)	1.20	2.83	0.070
Moon phase (cyclic)	1.15	1.87	0.170
Precipitation	2.63	19.61	<0.001
Pipistrellus nathusii **— Season-specific circular wind-direction model (spring and autumn windows)**			
Day within seasonal window	2.32	1.38	0.165
Hours after sunset	6.88	186.46	<0.001
Temperature	6.37	47.07	<0.001
Wind speed × Spring	4.45	5.29	<0.001
Wind speed × Autumn	5.24	6.65	<0.001
Wind direction (cyclic) × Spring	1.86	9.56	0.007
Wind direction (cyclic) × Autumn	<0.01	<0.01	0.497

Smooth term	EDF	Chi-square	p
Moon phase (cyclic)	2.99	144.55	0.042
Precipitation	3.93	1.99	0.089
Calendar night (random intercept)	84.97	6.23	<0.001
***Pipistrellus nathusii* — Season-specific migration-relative tailwind/crosswind model (spring and autumn windows)**			
Day within seasonal window	2.72	1.10	0.236
Hours after sunset	6.88	172.77	<0.001
Temperature	6.38	46.31	<0.001
Tailwind component × Spring	3.34	6.68	<0.001
Tailwind component × Autumn	4.38	7.81	<0.001
Crosswind component × Spring	3.45	4.37	0.001
Crosswind component × Autumn	2.84	2.60	0.040
Moon phase (cyclic)	2.40	109.07	0.043
Precipitation	3.92	2.09	0.086
Calendar night (random intercept)	84.27	6.03	<0.001
***N. noctula/N. leisleri* — Full season, hourly counts (negative binomial, calendar-night random effect)**			
Day of year × Sarıyer Forest	8.42	14.22	<0.001
Day of year × Atatürk City Forest	6.94	18.16	<0.001
Day of year × ITU Campus	7.03	7.34	<0.001
Day of year × Yıldız Park	5.19	4.58	<0.001
Hours after sunset	6.76	60.57	<0.001
Temperature	3.65	13.19	<0.001
Wind speed	2.56	1.68	0.168
Wind direction (cyclic)	2.12	3.81	0.010
Moon phase (cyclic)	2.89	20.22	<0.001
Precipitation	2.43	1.01	0.315
Calendar night (random intercept)	70.32	1.17	<0.001
***N. noctula/N. leisleri* — Full season, presence/absence (binomial; sensitivity)**			
Day of year × Sarıyer Forest	8.05	106.31	<0.001
Day of year × Atatürk City Forest	6.60	112.60	<0.001
Day of year × ITU Campus	7.12	60.64	<0.001
Day of year × Yıldız Park	5.47	37.05	<0.001
Hours after sunset	6.52	292.48	<0.001
Temperature	3.27	42.42	<0.001
Wind speed	2.55	16.00	0.001
Wind direction (cyclic)	3.80	20.01	<0.001
Moon phase (cyclic)	2.08	8.99	0.004
Precipitation	2.19	13.74	0.004
***N. noctula/N. leisleri* — Season-specific circular wind-direction model (spring and autumn windows)**			
Day within seasonal window	2.61	2.80	0.085
Hours after sunset	6.73	57.02	<0.001
Temperature	4.66	14.05	<0.001
Wind speed × Spring	2.02	0.45	0.521
Wind speed × Autumn	1.00	<0.01	0.945

Smooth term	EDF	Chi-square	p
Wind direction (cyclic) × Spring	<0.01	<0.01	0.397
Wind direction (cyclic) × Autumn	2.86	6.39	0.025
Moon phase (cyclic)	2.93	33.89	<0.001
Precipitation	2.39	1.89	0.139
Calendar night (random intercept)	65.67	2.04	<0.001
***N. noctula*/*N. leisleri* — Season-specific migration-relative tailwind/crosswind model (spring and autumn windows)**			
Day within seasonal window	2.63	3.22	0.035
Hours after sunset	6.75	62.22	<0.001
Temperature	4.59	10.82	<0.001
Tailwind component × Spring	1.12	2.69	0.094
Tailwind component × Autumn	4.46	8.73	<0.001
Crosswind component × Spring	2.32	1.62	0.154
Crosswind component × Autumn	1.00	0.05	0.822
Moon phase (cyclic)	2.74	27.55	0.004
Precipitation	2.94	2.53	0.046
Calendar night (random intercept)	66.93	2.11	<0.001

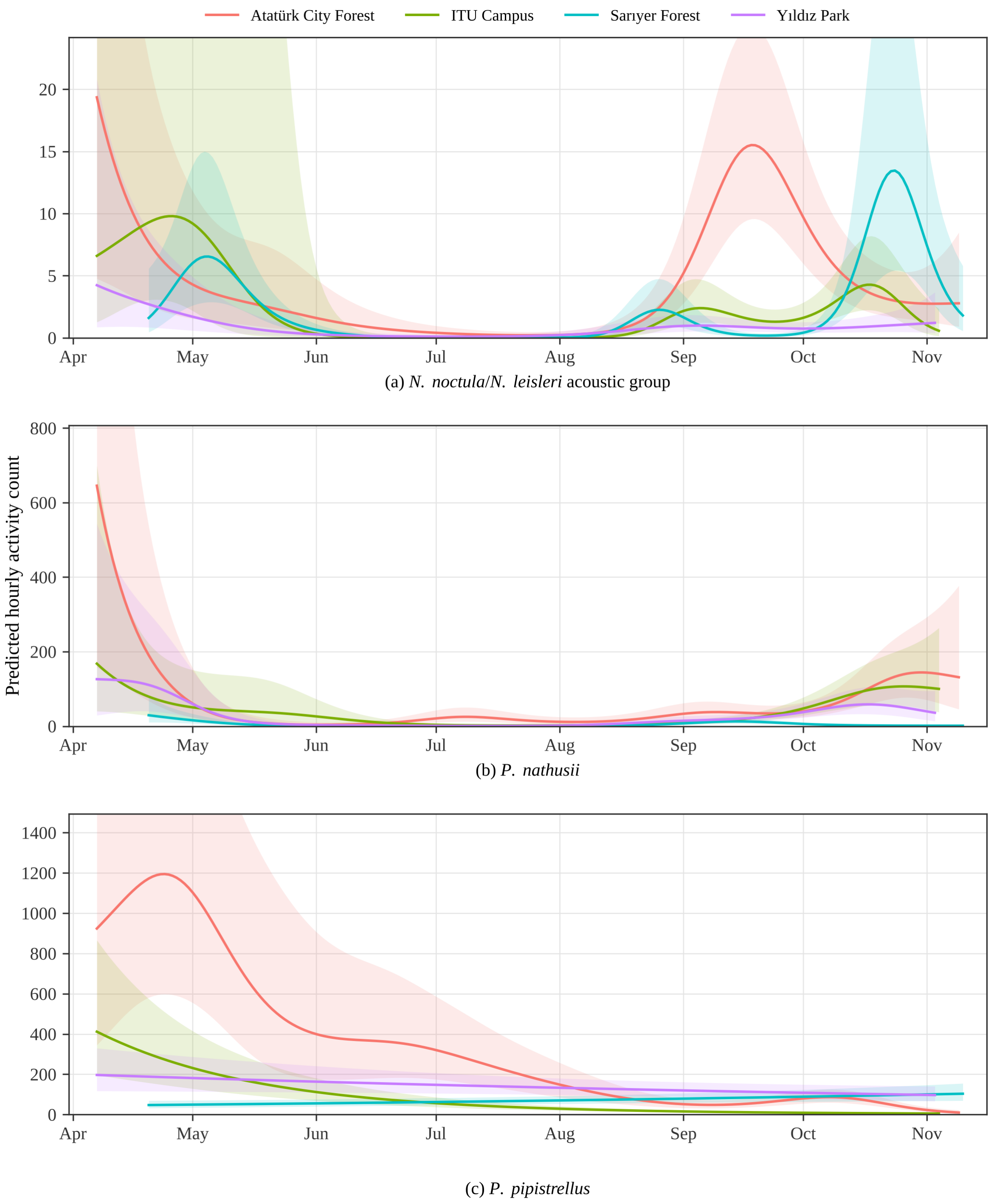

Figure S1. Site-specific seasonal activity of (a) the *Nyctalus noctula*/*N. leisleri* acoustic group, (b) *Pipistrellus nathusii* and (c) *P. pipistrellus* at the four monitoring stations. Lines show predicted hourly pass counts from the site-specific day-of-year smooths of the full-season negative-binomial GAMs with a calendar-night random effect (corrected weather data); other covariates are held at their station means, and the random effect is excluded from the predictions. Shaded ribbons are 95% confidence intervals; the vertical axis is truncated at 1.25 times the highest fitted value so that the autumn maxima remain visible, and ribbons may extend beyond the plotted range near the start of sampling. Predictions are shown only for the period sampled at each station. Note the different y-axis scales among panels.

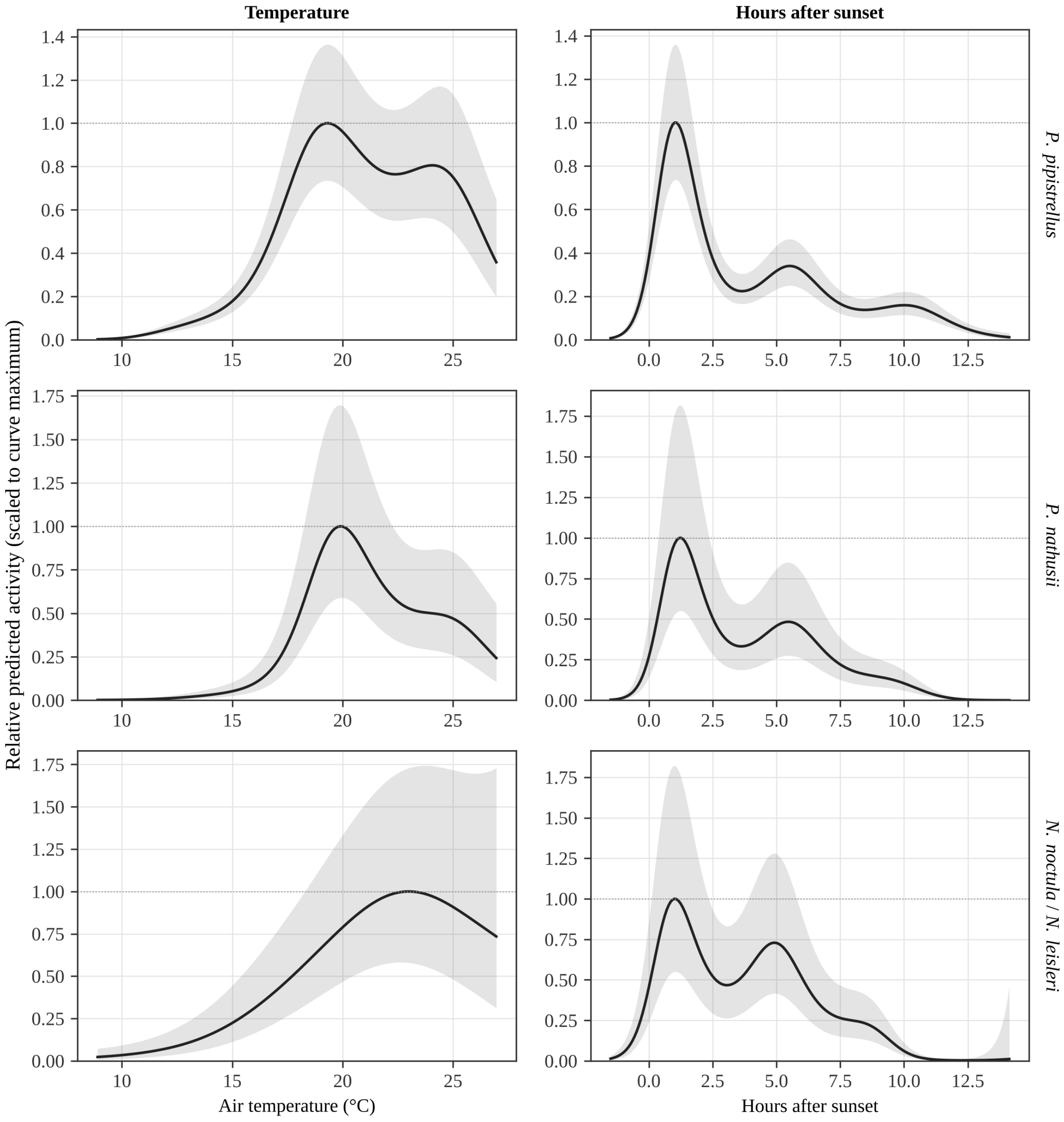

Figure S2. Partial effects of air temperature (left column) and hours after sunset (right column) on predicted hourly activity of *P. pipistrellus* (top), *P. nathusii* (middle) and the *N. noctula*/*N. leisleri* acoustic group (bottom) from the full-season negative-binomial GAMs with a calendar-night random effect. Predictions were made for each station with the other covariates at their station means and the random effect excluded, averaged over the four stations, and scaled to the maximum of each curve (dotted line = 1). Shaded ribbons are 95% confidence intervals.

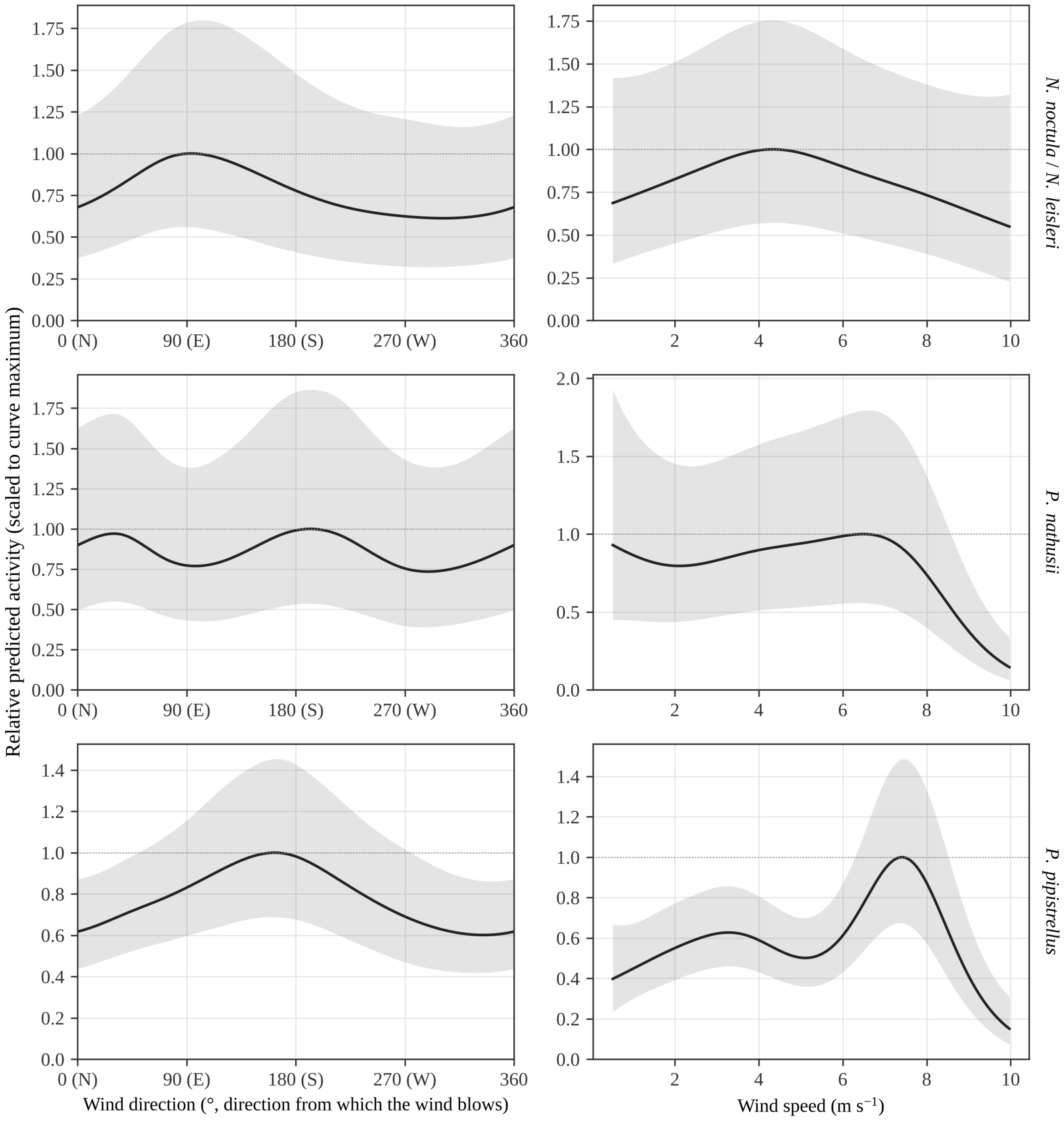

Figure S3. Partial effects of 10 m wind direction (left; degrees, meteorological convention, i.e. the direction from which the wind blows, modelled as a cyclic smooth) and 10 m wind speed (right; m s^{-1}) on predicted hourly activity of the *N. noctula*/*N. leisleri* acoustic group (top), *P. nathusii* (middle) and *P. pipistrellus* (bottom) from the full-season GAMs with a calendar-night random effect. Predictions were averaged over the four stations and scaled to the maximum of each curve as in Figure S2; shaded ribbons are 95% confidence intervals. Meteorological data are hourly NASA POWER estimates converted from UTC to local time (UTC+3). The wind-direction term was not significant for *P. nathusii* (Table S4).

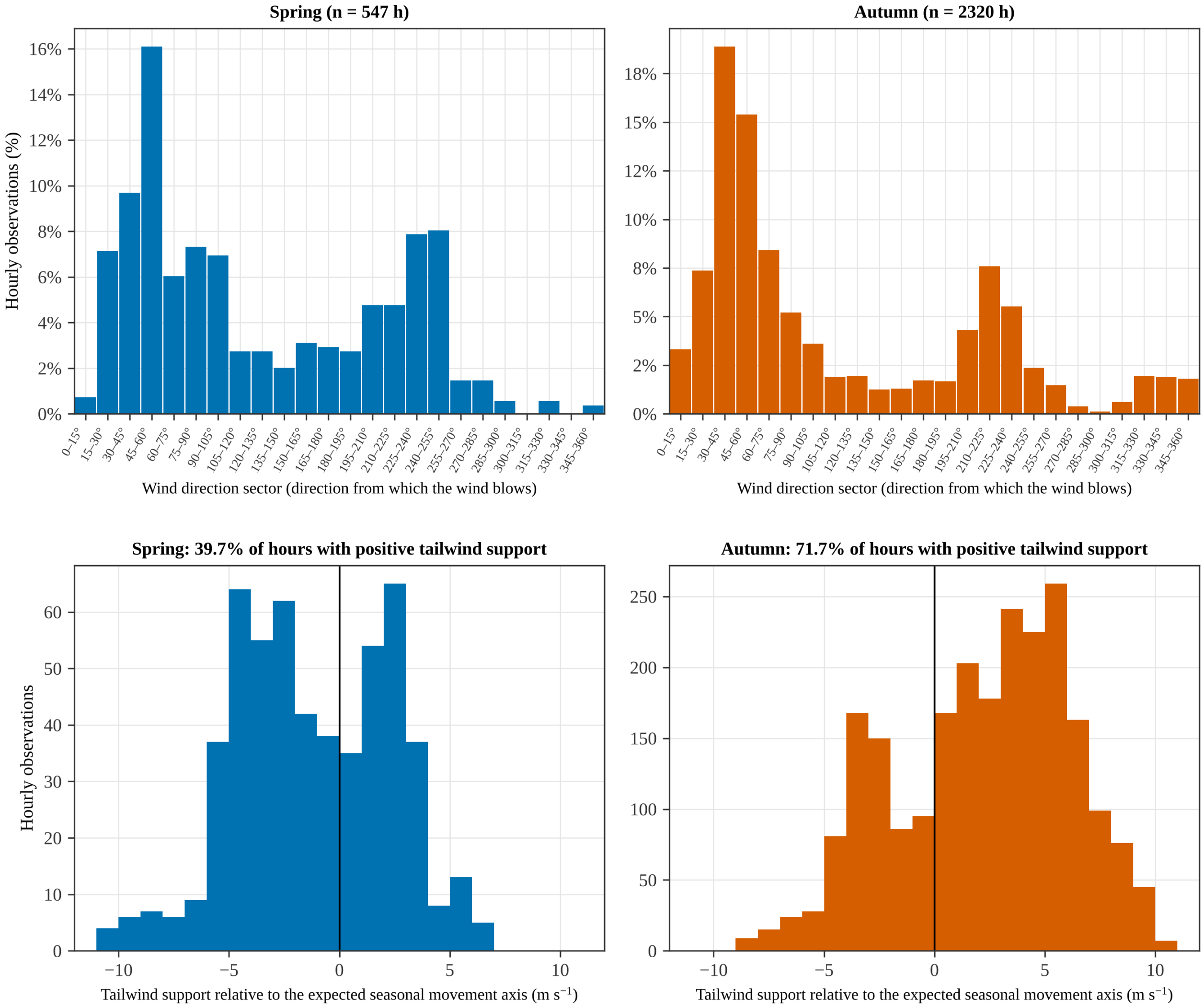

Figure S4. Wind conditions during the spring (7 April–31 May) and autumn (14 August–9 November 2022) windows (UTC+3). Upper panels: frequency distribution of hourly 10 m wind direction in 24 sectors of 15° (direction from which the wind blows). Lower panels: distribution of the tailwind component of the hourly wind vector relative to the assumed seasonal movement axis (spring: toward 45° NE; autumn: toward 225° SW). Positive values indicate winds supporting movement in the expected seasonal direction; the vertical line marks zero tailwind support.

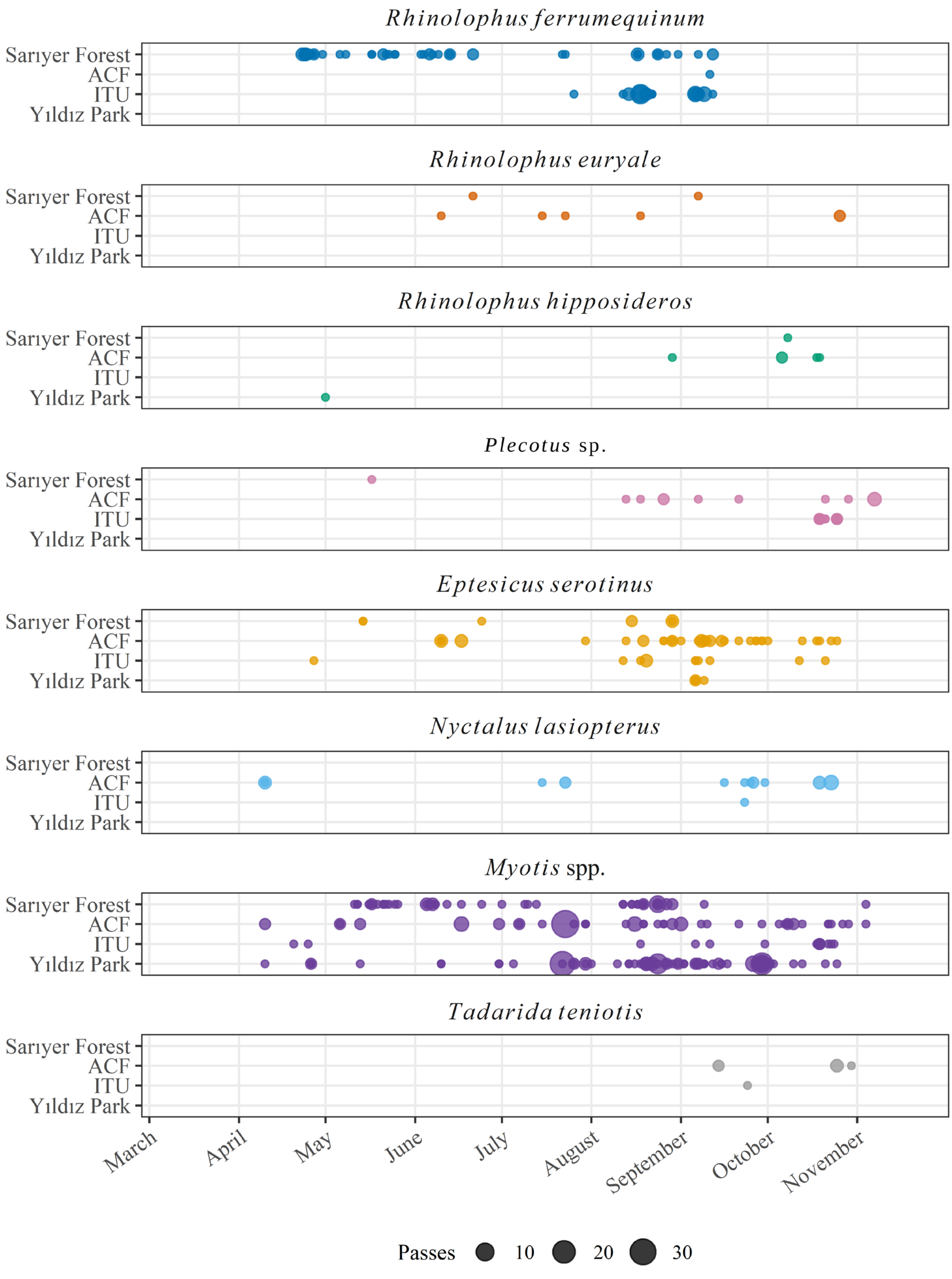

Figure S5. Nightly detections of less frequently recorded bat taxa at the four monitoring stations, April–November 2022. Each point is a recording night with at least one pass; point size is proportional to the number of passes recorded that night.

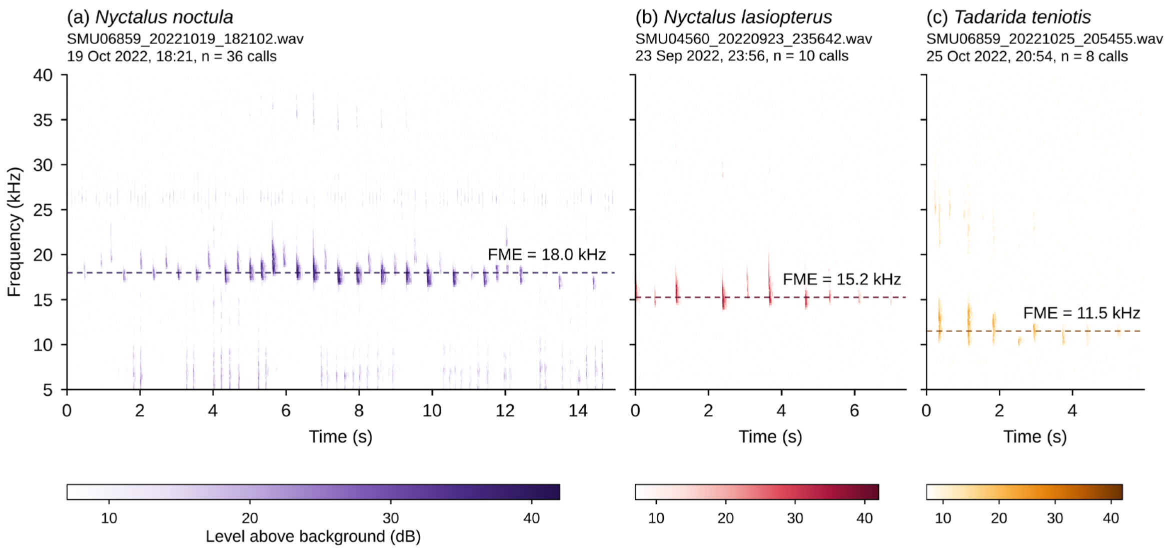

Figure S6. Example spectrograms of search-phase echolocation sequences of (a) *Nyctalus noctula* (Atatürk City Forest, 19 October 2022, 18:21; 36 calls), (b) *N. lasiopterus* (ITU Campus, 23 September 2022, 23:56; 10 calls), and (c) *Tadarida teniotis* (Atatürk City Forest, 25 October 2022, 20:54; 8 calls), illustrating the identification criteria described in Section 2.3 of the main text. Dashed lines show the median frequency of maximum energy (FME) of the calls in each sequence (18.0, 15.2, and 11.5 kHz, respectively). Spectrograms share the same frequency axis (5–40 kHz) and colour scale (level above background noise, dB); time axes differ according to recording length. The original file name is given above each panel.
